# Phase separation of SHP2^E76K^ promotes malignant transformation of mesenchymal stem cells by activating mitochondrial complexes

**DOI:** 10.1172/jci.insight.170340

**Published:** 2024-03-07

**Authors:** Chen Kan, Zhenya Tan, Liwei Liu, Bo Liu, Li Zhan, Jicheng Zhu, Xiaofei Li, Keqiong Lin, Jia Liu, Yakun Liu, Fan Yang, Mandy Wong, Siying Wang, Hong Zheng

**Affiliations:** 1 Department of Pathophysiology, School of Basic Medical Sciences, Stem Cell Regeneration Research Center, Anhui Medical University, Hefei, China.; 2 Department of Pathogen Biology and Immunology, School of Medical Technology, Anhui Medical College, Hefei, China.; 3 Department of Cell Center, 901st Hospital of PLA Joint Logistic Support Force, Anhui, Hefei, China.; 4 Department of Biological Sciences, Georgia Institute of Technology, Atlanta, Georgia, USA.

**Keywords:** Oncology, Stem cells, Adult stem cells, Cancer, Mouse stem cells

## Abstract

Mesenchymal stem cells (MSCs), suffering from diverse gene hits, undergo malignant transformation and aberrant osteochondral differentiation. Src homology region 2–containing protein tyrosine phosphatase 2 (SHP2), a nonreceptor protein tyrosine phosphatase, regulates multicellular differentiation, proliferation, and transformation. However, the role of SHP2 in MSC fate determination remains unclear. Here, we showed that MSCs bearing the activating SHP2^E76K^ mutation underwent malignant transformation into sarcoma stem-like cells. We revealed that the SHP2^E76K^ mutation in mouse MSCs led to hyperactive mitochondrial metabolism by activating mitochondrial complexes I and III. Inhibition of complexes I and III prevented hyperactive mitochondrial metabolism and malignant transformation of SHP2^E76K^ MSCs. Mechanistically, we verified that SHP2 underwent liquid-liquid phase separation (LLPS) in SHP2^E76K^ MSCs. SHP2 LLPS led to its dissociation from complexes I and III, causing their hyperactivation. Blockade of SHP2 LLPS by LLPS-defective mutations or allosteric inhibitors suppressed complex I and III hyperactivation as well as malignant transformation of SHP2^E76K^ MSCs. These findings reveal that complex I and III hyperactivation driven by SHP2 LLPS promotes malignant transformation of SHP2^E76K^ MSCs and suggest that inhibition of SHP2 LLPS could be a potential therapeutic target for the treatment of activated SHP2–associated cancers.

## Abstract 2

Liquid&ndash;liquid phase separation of SHP2 promotes malignant transformation of mesenchymal stem cells.

## Graphical abstract

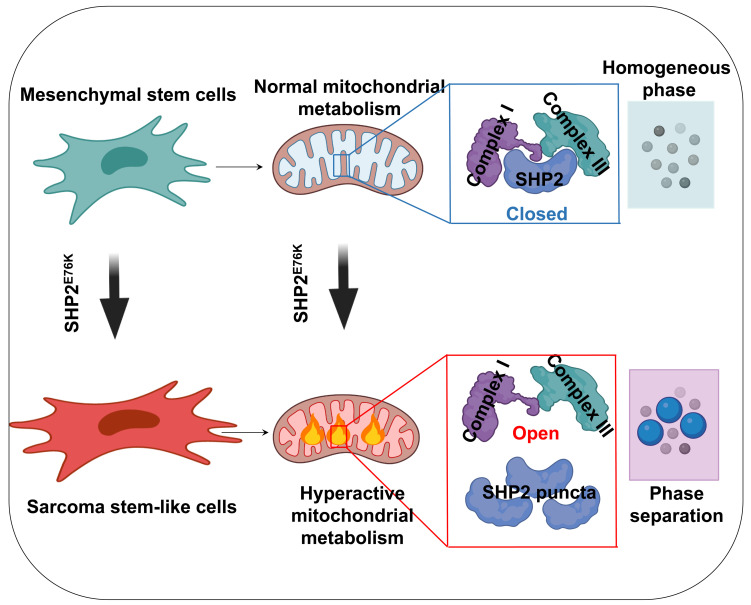

## Introduction

Src homology region 2–containing (SH2-containing) protein tyrosine phosphatase 2 (SHP2) is encoded by protein tyrosine phosphatase non-receptor type 11 (*PTPN11*). Under physiological conditions, SHP2 is autoinhibited and displays hardly any catalytic activity. However, activating mutant SHP2 structurally exposes the protein tyrosine phosphatase (PTP) domain so that it can dephosphorylate downstream proteins or act as a signaling adaptor protein and consequently trigger oncogenic signaling cascades, such as RAS/ERK, JNK, mTOR, PI3K/AKT, and TP53 (1–4). SHP2 gain-of-function (GOF) mutations, such as E76K and D61G mutations, are closely associated with numerous diseases, such as Noonan syndrome and associated cancers (4, 5). Although GOF mutations in SHP2 have been found in many diseases (6), the underlying mechanism remains unclear.

Mesenchymal stem cells (MSCs), also called multipotent stromal cells and mesenchymal stromal cells, can differentiate along multiple connective lineages (4, 7). Since 1970, MSCs were initially identified by Friedenstein and colleagues in rodent bone marrow and then were found in many other tissues with divergence in phenotype and specialization (7). They are essential for the development, maintenance, function, and regeneration of most tissues. However, when MSCs are challenged by devastating genetic mutations, homeostasis is disrupted, and they undergo aberrant fate determination (8–16). MSCs can be hit by activin receptor type-1 (*ACVR1*) and *GNAS* and abnormally differentiate into ectopic bones in soft tissues (8, 9). Additionally, increasing evidence suggests that MSCs might be the cellular origin of sarcoma, and several types of sarcomas have been modeled by inducing transformation of MSCs with different oncogenic mutations (10–13, 15, 16). A previous study revealed that deletion of SHP2 in mouse MSCs causes metachondromatosis, suggesting that SHP2 determines MSC transformation (17). Moreover, GOF SHP2 mutations are commonly found in Noonan syndrome, which is characterized by a short stature (5). These clues inspired us to investigate the role of GOF SHP2 in MSC fate determination.

Metabolic dysregulation, including aberrant glycolysis and/or mitochondrial activity, is a hallmark of cancer initiation and progression, providing enough energy for malignant proliferation (18). Aerobic glycolysis is the prominent pathway that drives cancer development, including sarcoma, although oxygen is abundant (19). However, mitochondrial function is also required for cell transformation and proliferation (20). Recent studies have shown that oxidative phosphorylation (OXPHOS) can also be upregulated in certain cancers, including leukemias, lymphomas, pancreatic ductal adenocarcinoma, high-OXPHOS subtype melanoma, and endometrial carcinoma and that this can occur even in the face of active glycolysis (21, 22). Mitochondrial respiratory complexes, including complexes I, II, III, IV, and V, are pivotal for ATP production and mitochondrial metabolism (23–25). Upregulation of complex activity is observed in several cancers, and targeting complexes with molecular inhibitors could attenuate malignancy (22, 26). Mutations in key enzymes of OXPHOS, such as isocitrate dehydrogenase (IDH), succinate dehydrogenase (SDH), and fumarate hydratase, promote cancer initiation and development, including sarcoma (18, 27, 28). However, the alteration of mitochondrial metabolism in MSCs harboring GOF SHP2 mutations is largely unknown.

Liquid-liquid phase separation (LLPS), also called condensation of biomolecules, has been reported to be involved in various biological processes, including tumorigenesis (29). Phase separation–induced biomolecular condensates form coherent structures that can compartmentalize and concentrate biochemical reactions within cells (30, 31). Thus, LLPS of proteins can regulate myriad cellular functions associated with cancer pathophysiology, such as nuclear function control of oncogenes, cellular quality regulation, and spatiotemporal organization of biochemical pathways, including metabolic activities (32, 33). However, the detailed mechanism by which condensates shape the biochemical reactions of cancer cells remains elusive. Our previous study revealed that SHP2^E76K^ promoted OXPHOS in mouse embryo fibroblasts and underwent LLPS (2, 34). Therefore, it is important to investigate the role of SHP2 LLPS in metabolic regulation in GOF-mutant SHP2-associated diseases.

In the present study, we showed that the GOF SHP2^E76K^ mutation led to malignant transformation of MSCs but did not affect their trilineage differentiation capacity. We found that MSCs isolated from SHP2^E76K^ mutation mice showed hyperactive mitochondrial metabolism, including hyperactive complexes I and III. Inhibition of complexes I and III prevented hyperactive mitochondrial metabolism and malignant transformation of SHP2^E76K^ MSCs. Furthermore, we revealed that SHP2^E76K^ underwent LLPS and dissociated from complexes I and III in MSCs, which led to complex I and III hyperactivation. Moreover, blockade of SHP2 LLPS by specific LLPS-defective mutations and allosteric inhibitors prevented complex I and III activation and malignant transformation of SHP2^E76K^ MSCs. Thus, our study provides a potential mechanism for GOF-mutant SHP2-associated diseases and further bridges the gap between LLPS and malignant transformation of MSCs, as well as suggests that complexes I and III and SHP2 LLPS could be the therapeutic targets for the treatment of SHP2-activating mutation-associated tumors.

## Results

### MSCs harboring the SHP2^E76K^ mutation undergo malignant transformation and initiate sarcomagenesis in mice.

To better understand how SHP2 GOF mutations may contribute to mouse MSC fate determination, we isolated bone marrow MSCs from Mx1-Cre and Mx1-Cre SHP2^E76K^ mice that we developed in the present study (Supplemental Figure 1A; supplemental material available online with this article; https://doi.org/10.1172/jci.insight.170340DS1). We selected Mx1^+^ cells over Nestin^+^ or Gli1^+^ MSCs, as previous studies have shown that these cells are more frequent in bone marrow (35, 36). In addition, our finding of a *neo* cassette deletion in SHP2^E76K^ MSCs verified successful Mx1-Cre–mediated gene recombination (Supplemental Figure 1B). PTP assays also supported that SHP2 was successfully activated in SHP2^E76K^ MSCs (Supplemental Figure 1C). Moreover, the expression of SHP2 in Mx1-Cre mouse MSCs was identical to that in Mx1-Cre SHP2^E76K^ mouse MSCs (Supplemental Figure 1D). Flow cytometry revealed that both WT and SHP2^E76K^ MSCs expressed the anticipated marker molecules for MSCs, including PDGFRα (CD140a), CD105, and Sca1; note that no markers of hematopoietic (i.e., CD11b and CD45) or endothelial cells (i.e., CD31) were detected in the isolated WT and SHP2^E76K^ MSCs (Supplemental Figure 2, A and B).

MSCs usually display trilineage differentiation potential, including adipogenesis, chondrogenesis, and osteogenesis (7). Therefore, we first compared differences in trilineage differentiation capacity between SHP2^E76K^ and WT MSCs. We performed trilineage differentiation assays (37) and found no significant difference in trilineage differentiation potential between WT and SHP2^E76K^ MSCs (Supplemental Figure 3, A–F).

**Figure 1:**
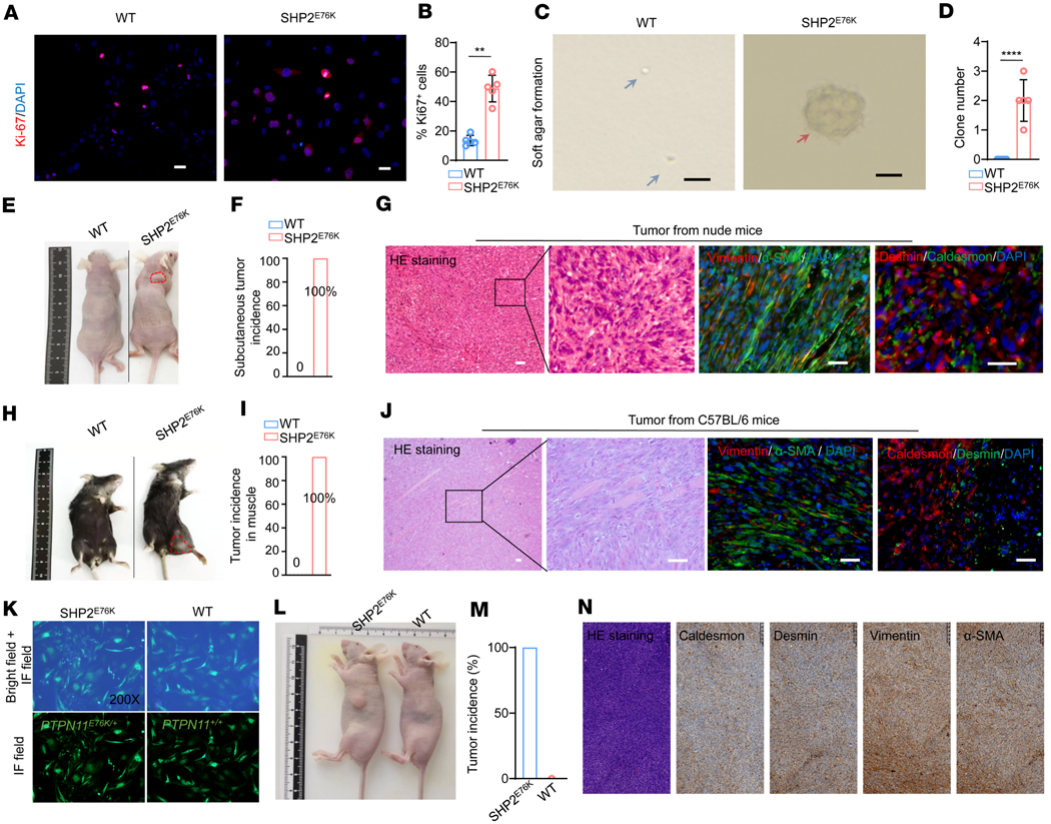
MSCs with the SHP2^E76K^ mutation undergo malignant transformation and model leiomyosarcoma formation. (**A** and **B**) immunofluorescence (IF) images (**A**) and statistical analysis (**B**) of Ki67 staining of WT and SHP2^E76K^ MSCs (*n* = 5 per group). Scale bar, 200 μm. Data are represented as the mean ± SD. **P < 0.01 (2-tailed unpaired *t* test). (**C** and **D**) Microscopy image (**C**) and statistical analysis (**D**) of soft agar colony formation capacity of WT and SHP2^E76K^ MSCs (*n* = 5 per group). Scale bar, 200 μm. Data are represented as the mean ± SD. ****P < 0.01 (2-tailed unpaired *t* test). (**E**) Xenograft tumor transplantation of WT and SHP2^E76K^ MSCs in nude mice (*n* = 5 per group). (**F**) Statistical analysis of the tumor incidence of WT and SHP2^E76K^ MSCs in nude mice (*n* = 5). (**G**) HE staining and IF staining images of the indicated antibodies in SHP2^E76K^-induced sarcoma from nude mice. Scale bar, 200 μm. (**H**) Xenograft tumor transplantation of WT and SHP2^E76K^ MSCs in immunocompetent (C57BL/6) mice. (**I**) Statistical analysis of the tumor incidence of WT and SHP2^E76K^ MSCs in C57BL/6 mice (*n* = 4 per group). (**J**) HE staining and IF staining of the indicated antibodies in SHP2^E76K^-induced sarcoma from C57BL/6 mice. Scale bar, 200 μm. (**K**) Representative IF images of WT and SHP2^E76K^ human umbilical cord MSCs in the field of 200×. (**L** and **M**) Representative tumor images (**L**) and statistical analysis (**M**) of tumor incidence in nude mice with subcutaneous inoculation of WT and SHP2^E76K^ human MSCs (*n* = 5 per group). (**N**) Representative HE and immunohistochemical images of tumor developed by SHP2^E76K^ human umbilical cord MSCs. Scale bar, 500 μm.

Since GOF SHP2 did not impair mouse MSC trilineage differentiation in vitro, we next compared differences in growth between SHP2^E76K^ and WT MSCs. We performed Ki67 staining, which revealed that MSCs expressing SHP2^E76K^ had a significantly increased capacity to proliferate compared with WT MSCs (Figure 1, A and B). Considering the oncogenic effect of GOF SHP2^E76K^, we speculated that SHP2^E76K^-induced hyperproliferation could be associated with malignant transformation of MSCs. We next investigated whether these cells were malignant by examining their capacity for nonanchored growth, a common behavior for determining malignancy. Soft agar colony formation assays showed that, unlike WT MSCs, SHP2^E76K^ MSCs acquired the capacity for nonanchored growth (Figure 1, C and D), suggesting that SHP2^E76K^ MSCs underwent malignant transformation. To determine whether SHP2^E76K^ MSCs could initiate tumorigenesis in vivo, we subcutaneously injected WT and SHP2^E76K^ MSCs into nude mice and found that SHP2^E76K^ MSCs but not WT MSCs could form tumors in nude mice (Figure 1, E and F). Hematoxylin and eosin (HE) staining verified that SHP2^E76K^ MSCs can form soft tissue sarcoma in nude mice (Figure 1G). GOF mutations of SHP2 were found in rhabdomyosarcoma (6, 38). Thus, we performed IF staining of tumor sections to monitor the accumulation of rhabdomyosarcoma (RMS) markers, including MyoD1 and myogenin. However, no positive signals were detected, suggesting that SHP2^E76K^ mouse bone marrow MSCs could not initiate the formation of RMS in the subcutaneous site of nude mice (Supplemental Figure 4A). Previous studies showed that bone marrow MSCs could initiate leiomyosarcoma formation in vivo (15). Therefore, we again performed IF staining of tumor sections to examine the expression of leiomyosarcoma markers, including vimentin, α–smooth muscle actin (α-SMA), caldesmon, and desmin, at the xenograft site, with the detected signals supporting these as leiomyosarcoma (Figure 1G and Supplemental Figure 4B).

Moreover, since RMS is a malignant cancer that develops from muscles, we injected SHP2^E76K^ MSCs into the tibial muscle of C57BL/6 mice following a previously reported approach for inducing xenograft RMS tumors (39). Only SHP2^E76K^ MSCs could form tumors (Figure 1, H and I). In addition to leiomyosarcoma formation in nude mice mentioned above, tibial muscle sarcoma in C57BL/6 mice initiated by SHP2^E76K^ MSCs also expressed leiomyosarcoma markers (Supplemental Figure 4C). HE and IF staining supported that these putative leiomyosarcomas developed in muscle tissue (Figure 1J). However, IF staining of tumor sections revealed that the tumor cells did not express MyoD1 and myogenin, markers of RMS cells (40), suggesting that this inconsistency between clinical SHP2 GOF mutation in patients and mouse sarcomagenesis could be associated with the origin and species of MSCs (Supplemental Figure 4D).

Long-term culture has been reported as a potential inducer for the malignant transformation of MSCs (41). Excluding a potential impact of culture time on the malignant transformation of MSCs, we performed Ki67 and β-galactosidase (β-gal) staining to detect the proliferation of WT and SHP2^E76K^ MSCs following successive passages. Obviously, WT MSCs following successive passages were senescent rather than undergoing malignant transformation, evidenced by a significant increase of β-gal^+^ cells and a significant decrease of Ki67^+^ cells in SHP2^E76K^ MSCs, compared with that in WT MSCs at passage 10 (Supplemental Figure 5, A–D).

Of note, like the sarcomagenesis in WT mice injected with SHP2^E76K^ MSCs, SHP2^E76K^ MSCs can also initiate sarcomagenesis in Mx1-Cre SHP2^E76K^ mice (Supplemental Figure 6, A–C). Additionally, WT mice with subcutaneous inoculation of SHP2^E76K^ MSCs underwent lung metastasis (Supplemental Figure 6D). Moreover, human umbilical cord MSCs that were transfected with *PTPN11^E76K/+^* plasmid could develop leiomyosarcoma in nude mice (Figure 1, K–N). Taken together, these results indicate that MSCs harboring the SHP2^E76K^ mutation can undergo malignant transformation and initiate sarcomagenesis.

### A subpopulation of sarcoma stem-like cells exists among SHP2^E76K^ MSCs.

**Figure 2:**
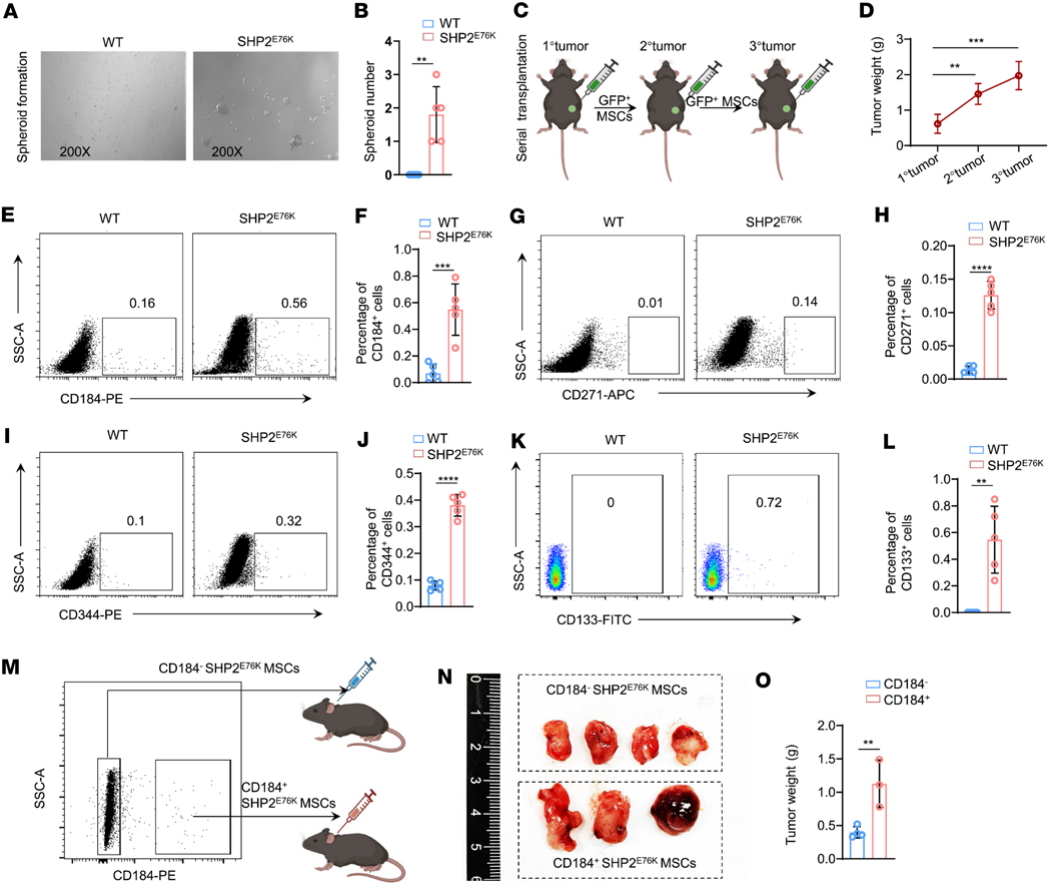
MSCs with the SHP2^E76K^ mutation transform into sarcoma stem-like cells. (**A** and **B**) Representative images (**A**) and statistical analysis (**B**) of microspheres in WT and SHP2^E76K^ MSCs (*n* = 5 per group). Data are represented as the mean ± SD. **P < 0.01 (2-tailed unpaired *t* test). (**C**) Schematic outline of the study design, depicting the workflow of serial transplantation of SHP2^E76K^ MSCs. (**D**) Statistical analysis of tumor weight following serial transplantation of SHP2^E76K^ MSCs (*n* = 4 per group). Data are represented as the mean ± SD. **P < 0.01, ***P < 0.001 (2-tailed unpaired *t* test). (**E** and **F**) Representative flow cytometry images (**E**) and statistical analysis (**F**) of CD184-positive cells in WT and SHP2^E76K^ MSCs (*n* = 5 per group). Data are represented as the mean ± SD. ***P < 0.001 (2-tailed unpaired *t* test). (**G** and **H**) Representative flow cytometry images (**G**) and statistical analysis (**H**) of CD271-positive cells in WT and SHP2^E76K^ MSCs (*n* = 5 per group). Data are represented as the mean ± SD. ****P < 0.0001 (2-tailed unpaired *t* test). (**I** and **J**) Representative flow cytometry images (**I**) and statistical analysis (**J**) of CD344-positive cells in WT and SHP2^E76K^ MSCs (*n* = 5 per group). Data are represented as the mean ± SD. ****P < 0.0001 (2-tailed unpaired *t* test). (**K** and **L**) Representative flow cytometry images (**K**) and statistical analysis (**L**) of CD133-positive cells in WT and SHP2^E76K^ MSCs (*n* = 5 per group). Data are represented as the mean ± SD. **P < 0.01 (2-tailed unpaired *t* test). (**M**) Gating strategy for CD184-positive and CD184-negative SHP2^E76K^ MSCs’ isolation using FACS. (**N** and **O**) Representative tumor images (**N**) and statistical analysis of tumor (**O**) generated by CD184-positive and CD184-negative SHP2^E76K^ MSCs (*n* = 3 or 4 per group). Data are represented as the mean ± SD. **P < 0.01 (2-tailed unpaired *t* test).

In many cancers, tumors originate from the transformation of normal stem cells, resulting from the introduction of mutations in regulatory or signaling pathways that control the self-renewal of stem cells, resulting in the formation of cancer stem-like cells (42). To explore the potential transformation of SHP2^E76K^ MSCs into sarcoma stem-like cells, we first performed tumor spheroid formation assays. SHP2^E76K^ MSCs, but not WT MSCs, formed spheroids in culture (Figure 2, A and B). Cancer stem cells are identified by their transplantable tumor-initiating property (43). Validating that SHP2^E76K^ MSCs could form sarcoma stem-like cells in vivo, we further transfected a ZsGreen reporter gene with a lentiviral vector in SHP2^E76K^ MSCs (Supplemental Figure 7, A–D) and traced them in transplantable tumors following subcutaneous injection of ZsGreen-labeled SHP2^E76K^ MSCs in C57BL/6 mice. ZsGreen-labeled SHP2^E76K^ MSCs were subcutaneously injected into C57BL/6 mice (first recipients) (Figure 2C). Twenty-one days after implantation, the sarcomas were collected, and ZsGreen-labeled SHP2^E76K^ cells within them were isolated via a fluorescence-activated cell sorting (FACS) assay and were then used for a second round of implantation (second recipients) in the same cell counts as the first recipients (Figure 2C). After an additional 21 days, the above procedure was performed again in the third recipients. We found that sarcoma weights progressively increased following serial transplantation (Figure 2D).

We next examined the expression of the classic sarcoma stem cell markers, including CD184, CD271, CD344, and CD133 (44, 45). Interestingly, whereas WT MSCs expressed only the established MSC marker S100A4 (46), SHP2^E76K^ MSCs expressed CD184, CD271, CD344, and CD133 (Figure 2, E–L, and Supplemental Figure 8, A and B). Supporting our suppositions about sarcoma stem-like cells’ existence, flow cytometry analysis revealed that the proportion of CD184^+^, CD271^+^, CD344^+^, or CD133^+^ SHP2^E76K^ MSCs was significantly higher than that of WT MSCs (Figure 2, E–L). Moreover, to verify the stemness of these cells, we isolated CD133^–^, CD133^+^, CD184^–^, CD184^+^, CD271^–^, CD271^+^, CD344^–^, and CD344^+^ MSCs in the SHP2^E76K^ group and performed the in vivo tumor growth assay. Interestingly, among SHP2^E76K^ MSCs, CD184^+^ subtypes possessed the enhanced property to form tumor, compared with the CD184^–^ subtypes (Figure 2, M–O). However, no significant difference was observed between CD133^–^ and CD133^+^ subtypes, CD271^–^ and CD271^+^ subtypes, or CD344^–^ and CD344^+^ subtypes (Supplemental Figure 8, C–F). Collectively, these results show that SHP2^E76K^ MSCs can transform into sarcoma stem-like cells.

### SHP2^E76K^ mutation in MSCs leads to hyperactive mitochondrial metabolism.

**Figure 3:**
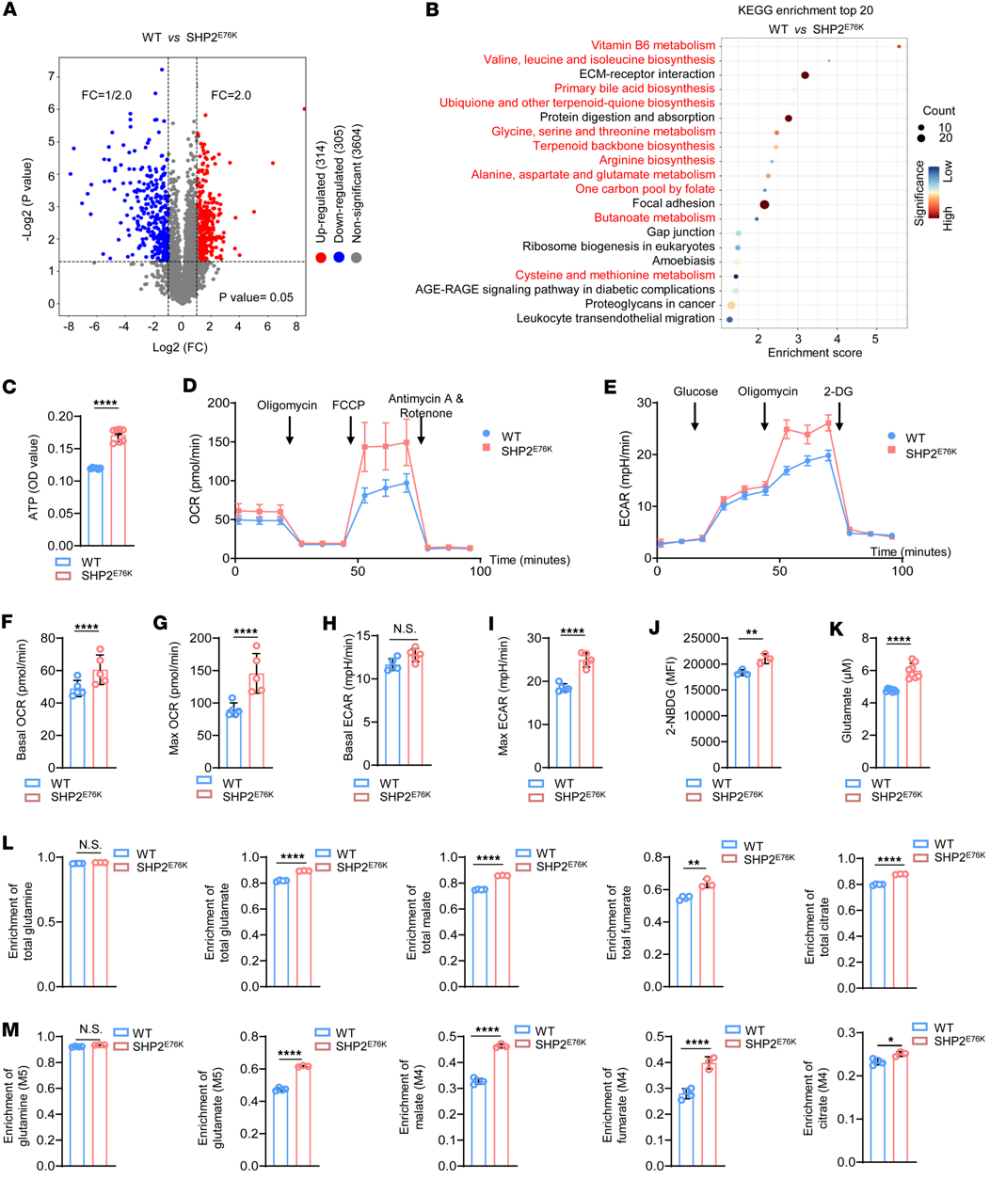
SHP2^E76K^ mutation causes hyperactive mitochondrial metabolism in MSCs. (**A**) Volcano plot analysis of WT and SHP2^E76K^ MSCs (*n* = 4). (**B**) KEGG pathway analysis of differentially expressed proteins (DEPs) of WT and SHP2^E76K^ MSCs. (**C**) Statistical analysis of ATP production in WT and SHP2^E76K^ MSCs (*n* = 6–7 per group). Data are represented as the mean ± SD. ****P < 0.0001 (2-tailed unpaired *t* test). (**D**) Representative image of OCR analysis in WT and SHP2^E76K^ MSCs. (**E**) Representative image of ECAR analysis in WT and SHP2^E76K^ MSCs. (**F** and **G**) Statistical analysis of mitochondrial respiration of WT and SHP2^E76K^ MSCs (*n* = 5 per group) at basal (**F**) and maximum (**G**) levels. Data are represented as the mean ± SD. ****P < 0.0001 (2-tailed unpaired *t* test). (**H** and **I**) Statistical analysis of the glycolytic function of WT and SHP2^E76K^ MSCs (*n* = 5 per group) at basal (**H**) and maximum (**I**) levels. Data are represented as the mean ± SD. ****P < 0.0001 (2-tailed unpaired *t* test). (**J** and **K**) Statistical analysis of glucose consumption (**J**, *n* = 3 per group) and glutamate content (**K**, *n* = 7 or 8 per group) in WT and SHP2^E76K^ MSCs. Data are represented as the mean ± SD. **P < 0.01, ****P < 0.0001 (2-tailed unpaired *t* test). (**L**) Statistical analysis of total enrichment of glutamine, glutamate, malate, fumarate, and citrate in WT and SHP2^E76K^ MSCs (*n* = 3 or 4 per group). Data are represented as the mean ± SD. **P < 0.01, ****P < 0.0001 (2-tailed unpaired *t* test). (**M**) Statistical analysis of the enrichment of glutamine (M5), glutamate (M5), malate (M4), fumarate (M4), and citrate (M4) in WT and SHP2^E76K^ MSCs (*n* = 3 or 4 per group). Data are represented as the mean ± SD. *P < 0.05, ****P < 0.0001 (2-tailed unpaired *t* test).

We next conducted proteomics assays to explore the potential mechanisms driving the observed malignant transformation of MSCs carrying the SHP2^E76K^ mutation. Principal component analysis of the proteomics data readily separated temporal trends in the expression profiles of the WT and SHP2^E76K^ MSCs (Supplemental Figure 9A). Comparison of WT and SHP2^E76K^ MSCs revealed a total of 314 upregulated differentially expressed proteins (DEPs) and 305 downregulated DEPs in SHP2^E76K^ MSCs (Figure 3A). Kyoto Encyclopedia of Genes and Genomes (KEGG) analysis of these DEPs revealed that 11 of the top 20 pathways enriched in SHP2^E76K^ MSCs were associated with energy metabolism (Figure 3B). Moreover, trend analysis of proteomics time series data revealed that energy metabolism–associated DEPs were randomly divided into 20 clusters. Note that energy metabolism–associated DEPs in 10 clusters were enriched in SHP2^E76K^ MSCs, whereas cluster 4 DEPs were enriched in WT MSCs, suggesting that metabolic dysfunction could be induced by the SHP2^E76K^ mutation in MSCs (Supplemental Figure 9, B–V).

ATP production can truly reflect the level of cellular energy metabolism (2). We assessed ATP production in WT and SHP2^E76K^ MSCs and found that SHP2^E76K^ MSCs generated more ATP than WT MSCs (Figure 3C). ATP can be generated by mitochondrial OXPHOS and glycolysis (22). Therefore, we performed Seahorse metabolic analysis of WT and SHP2^E76K^ MSC cultures and found that SHP2^E76K^ MSCs exhibited significantly higher mitochondrial OXPHOS (measured by oxygen consumption rate [OCR]) but not glycolysis (measured by extracellular acidification rate [ECAR]) than their WT counterparts, suggesting that mitochondrial OXPHOS could be the origin of higher ATP production in SHP2^E76K^ MSCs (Figure 3, D–F and H). Supporting this idea, lactate production (an indicator of glycolysis) in SHP2^E76K^ MSCs showed no obvious differences when compared to WT MSCs (Supplemental Figure 10A). In addition, SHP2^E76K^ MSCs had a significantly increased maximal respiratory capacity and glycolytic capacity compared with WT MSCs (Figure 3, D, E, G, and I), which indicated the metabolic plasticity of cancer cells upon environmental challenges (47). These results led us to reason that the hyperactive mitochondrial metabolism observed in SHP2^E76K^ MSCs was likely related to elevated glucose and amino acid consumption, which are the major energy resources for cells. To test this possibility, we conducted 2-NBDG assays, which revealed that SHP2^E76K^ MSCs exhibited significantly higher glucose consumption than WT MSCs (Figure 3J), and we found that the level of glutamate, a fundamental source of amino acid skeletons, was also significantly increased in SHP2^E76K^ MSCs (Figure 3K). Moreover, we performed the C^13^ isotope–labeled glutamine tracing assay in WT and SHP2^E76K^ MSCs and found that total C^13^-labeled glutamate, malate, fumarate, and citrate in SHP2^E76K^ MSCs were higher than in WT MSCs (Figure 3L). Moreover, the enrichment of glutamate (M5), malate (M4), fumarate (M4), and citrate (M4) in SHP2^E76K^ MSCs was also higher than that in WT MSCs (Figure 3M). There were no differences in branched chain amino acid (BCAA) levels between the WT and SHP2^E76K^ MSCs (Supplemental Figure 10B). Additionally, using the aforementioned DEPs, we performed gene set enrichment analysis and found that the genes annotated with mitochondrial functions, including mitochondrial organization, translation, and complex I, were enriched in SHP2^E76K^ MSCs (Supplemental Figure 10, C–G). Together, these results suggest that hyperactive mitochondrial metabolism could be a potential driver of the malignant transformation of SHP2^E76K^ MSCs.

### SHP2^E76K^ mutation in MSCs induces mitochondrial complex I and III hyperactivation.

Based on our above metabolic analysis, which revealed hyperactive mitochondrial metabolism, but not glycolysis, during the malignant transformation of SHP2^E76K^ MSCs, we next examined the mechanisms underlying this hyperactive mitochondrial metabolism. Mitochondrial metabolism is primarily dependent on mitochondrial DNA expression, biogenesis, and respiratory chain complex functions (48, 49). We first checked the mitochondrial DNA content, measured by relative expression of the cytochrome B (*CytB*) gene, and found no significant differences in *CytB* expression between WT and SHP2^E76K^ MSCs (Supplemental Figure 11A). We also examined mitochondrial mass through MitoTracker Green staining, which showed no obvious difference between WT and SHP2^E76K^ MSCs (Supplemental Figure 11, B and C).

**Figure 4:**
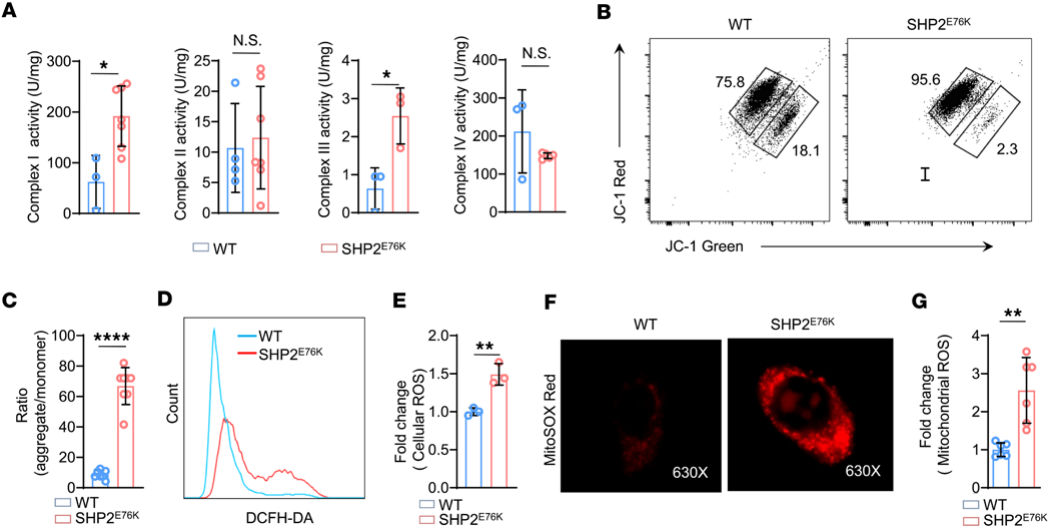
SHP2^E76K^ mutation leads to activation of mitochondrial complexes I and III in MSCs. (**A**) Statistical analysis of the activity of complexes I, II, III, and IV in WT and SHP2^E76K^ MSCs (*n* = 3 to 7 per group). Data are represented as the mean ± SD. *P < 0.05 (2-tailed unpaired *t* test). (**B** and **C**) Representative images (**B**) and statistical analysis (**C**) of MMP in WT and SHP2^E76K^ MSCs (*n* = 7 per group). Data are represented as the mean ± SD. ****P < 0.0001 (2-tailed unpaired *t* test). (**D** and **E**) Representative images (**D**) and statistical analysis (**E**) of ROS production in WT and SHP2^E76K^ MSCs (*n* = 3 per group). Data are represented as the mean ± SD. **P < 0.01 (2-tailed unpaired *t* test). (**F**) IF staining images of MitoSOX Red in WT and SHP2^E76K^ MSCs. (**G**) Statistical analysis of the relative level of mitochondrial ROS in WT and SHP2^E76K^ MSCs (*n* = 6 per group) via flow cytometry. Data are represented as the mean ± SD. **P < 0.01 (2-tailed unpaired *t* test).

We therefore speculated that mitochondrial complex function in MSCs could be altered following GOF SHP2 mutation. Since mitochondrial complexes I–IV together serve as the center of oxidative respiration (25), we checked the levels of complex I–IV subunits in MSCs with immunoblotting and examined their functions using mitochondrial complex activity kits. There were no differences in the protein levels between WT and SHP2^E76K^ MSCs for any of the 4 complexes (Supplemental Figure 11D). However, we found that complex I and III activity was significantly higher in SHP2^E76K^ MSCs than in WT MSCs (Figure 4A). Mitochondrial membrane potential (MMP) is generated by mitochondrial complexes, including complexes I and III (50). We observed that the MMP of SHP2^E76K^ MSCs was significantly increased over that of WT MSCs, verifying the hyperactivation of complexes I and III (Figure 4, B and C). Additionally, reactive oxygen species (ROS) are the byproducts of mitochondrial complex activation (51). DCFH-DA staining indeed showed a significant increase in total cellular ROS levels in MSCs harboring the SHP2^E76K^ mutation compared with WT MSCs (Figure 4, D and E). Moreover, IF staining with MitoSOX Red revealed that mitochondrial ROS levels in SHP2^E76K^ MSCs were also substantially higher than those in WT MSCs (Figure 4F). Further quantitative analysis of MitoSOX Red fluorescence using flow cytometry verified significant upregulation of mitochondrial ROS in SHP2^E76K^ MSCs compared with WT MSCs (Figure 4G). Overall, these results indicate that complex I and III hyperactivation could promote the hyperactive mitochondrial metabolism of SHP2^E76K^ MSCs.

### Pharmacological inhibition of mitochondrial complexes I and III in SHP2^E76K^ MSCs prevents hyperactive mitochondrial metabolism and malignant transformation.

**Figure 5:**
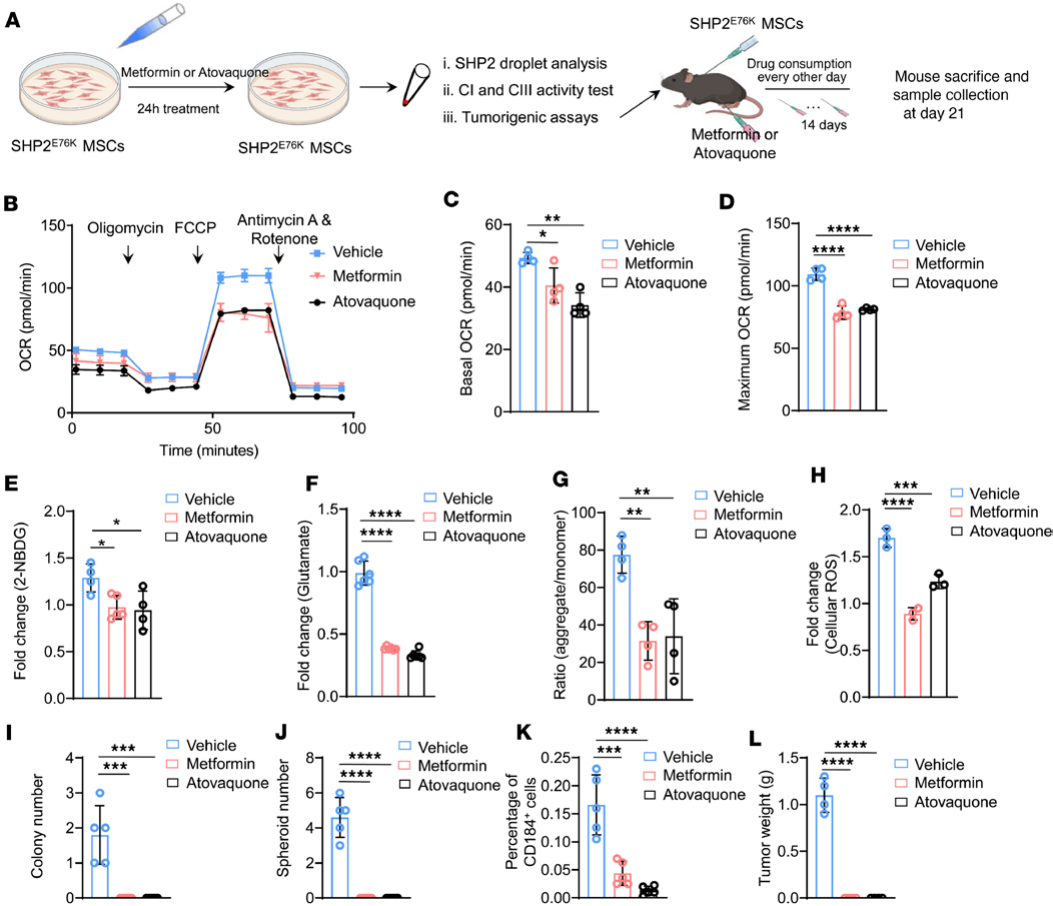
Inhibition of mitochondrial complexes I and III in SHP2^E76K^ MSCs prevents hyperactive mitochondrial metabolism and sarcomagenesis. (**A**) Schematic diagram depicting the effect of metformin and atovaquone on malignant transformation in SHP2^E76K^ MSCs. (**B**) Representative image of OCR analysis in SHP2^E76K^ MSCs treated with metformin and atovaquone. (**C** and **D**) Statistical analysis of mitochondrial respiration in SHP2^E76K^ MSCs at basal (**C**) and maximum levels (**D**) following treatment with metformin or atovaquone (*n* = 4 per group). Data are represented as the mean ± SD. *P < 0.05, **P < 0.01, ****P < 0.0001 (1-way ANOVA with multiple-comparison test). (**E**–**H**) Statistical analysis of glucose consumption (**E**), glutamate (**F**), MMP (**G**), and ROS (**H**) in SHP2^E76K^ MSCs (*n* = 4 or 5 per group) treated with metformin or atovaquone. Data are represented as the mean ± SD. *P < 0.05, **P < 0.01, ***P < 0.001, ****P < 0.0001 (1-way ANOVA with multiple-comparison test). (**I**) Statistical analysis of soft agar colony formation of SHP2^E76K^ MSCs treated with metformin or atovaquone (*n* = 5 per group). Data are represented as the mean ± SD. ***P < 0.001 (1-way ANOVA with multiple-comparison test). (**J**) Statistical analysis of spheroid formation of SHP2^E76K^ MSCs treated with metformin or atovaquone (*n* = 5 per group). Data are represented as the mean ± SD. ****P < 0.0001 (1-way ANOVA with multiple-comparison test). (**K**) Statistical analysis of percentage of CD184^+^ SHP2^E76K^ MSCs treated with metformin or atovaquone (*n* = 5 per group). Data are represented as the mean ± SD. ***P < 0.001, ****P < 0.0001 (1-way ANOVA with multiple-comparison test). (**L**) Statistical analysis of tumor formation of SHP2^E76K^ MSCs treated with metformin or atovaquone (*n* = 4 per group). Data are represented as the mean ± SD. ****P < 0.0001 (1-way ANOVA with multiple-comparison test).

Given our findings that the SHP2^E76K^ mutation induces malignant transformation of MSCs, and that this mutation substantially increases complex I and III activity in the mitochondria of SHP2^E76K^ MSCs, we explored whether pharmacologically disrupting complexes I and/or III could reduce the hyperactive mitochondrial metabolism and malignancy of MSCs bearing the SHP2^E76K^ mutation. Specifically, we separately administered 2 FDA-approved inhibitors of complexes I (metformin) and III (atovaquone) to SHP2^E76K^ MSCs. After initially verifying that metformin and atovaquone could selectively inhibit complex I and III activity in isolated mitochondria from SHP2^E76K^ MSCs (Supplemental Figure 12, A and B), we next investigated the effects of metformin and atovaquone on mitochondrial metabolism in SHP2^E76K^ MSCs (Figure 5A). Both metformin and atovaquone treatments led to significant attenuation of mitochondrial respiration (OCR) in SHP2^E76K^ MSCs at both baseline and maximum levels (Figure 5, B–D). Glucose consumption, glutamate content, MMP, and ROS levels were also significantly decreased in metformin- or atovaquone-treated SHP2^E76K^ MSCs (Figure 5, E–H).

To confirm the role of complexes I and III in the malignant transformation of SHP2^E76K^ MSCs, we pretreated SHP2^E76K^ MSCs with metformin and atovaquone (Figure 5A). These metformin- or atovaquone-treated SHP2^E76K^ MSCs were seeded onto soft agar. Both metformin and atovaquone significantly inhibited nonanchored growth of SHP2^E76K^ MSCs (Figure 5I and Supplemental Figure 12C). Moreover, metformin and atovaquone prevented the formation of spheroids of SHP2^E76K^ MSCs (Figure 5J and Supplemental Figure 12D). Additionally, metformin and atovaquone significantly decreased the population of CD184^+^ SHP2^E76K^ MSCs (Figure 5K and Supplemental Figure 12E). We further investigated whether metformin and atovaquone prevented sarcoma formation. These metformin- or atovaquone-treated SHP2^E76K^ MSCs were subcutaneously injected into C57BL/6 mice. On the same day, metformin and atovaquone were intraperitoneally (*i.p.*) injected into mice every other day for 2 weeks (Figure 5A). As expected, sarcomagenesis initiated from SHP2^E76K^ MSCs was inhibited by metformin and atovaquone treatments (Figure 5L and Supplemental Figure 12F). Together, these results demonstrate that hyperactivation of complexes I and III promotes the malignant transformation of SHP2^E76K^ MSCs.

### LLPS of SHP2 in MSCs underlies mitochondrial complex I and III hyperactivation.

**Figure 6:**
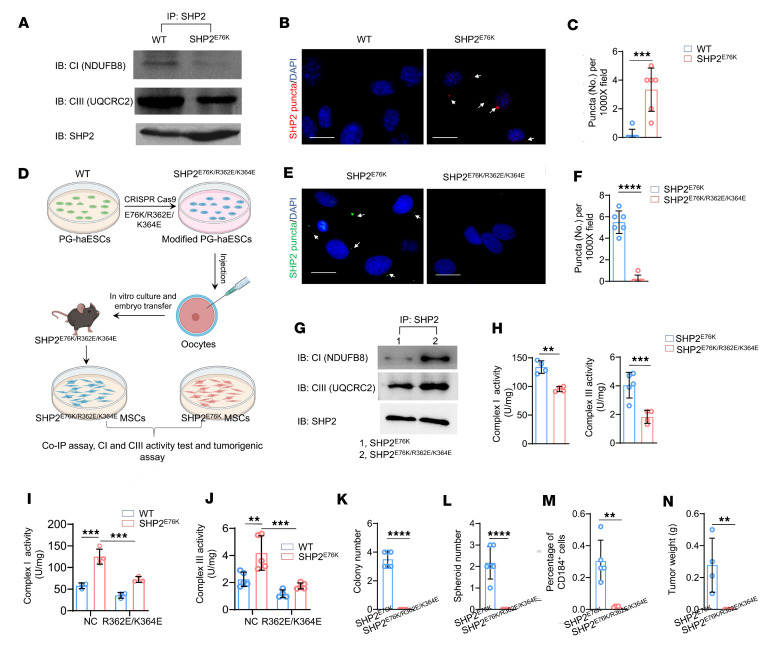
SHP2 LLPS promotes complex I and III hyperactivation. (**A**) WT and SHP2^E76K^ MSC lysates were examined by immunoprecipitation and immunoblotting as indicated. (**B** and **C**) Representative IF staining images (**B**) and statistical analysis (**C**) of SHP2 droplets in WT and SHP2^E76K^ MSCs (*n* = 6 per group). Data are represented as the mean ± SD. ***P < 0.001 (2-tailed unpaired *t* test). Scale bar, 15 μm. (**D**) Workflow of the establishment of a mouse model harboring a SHP2 triple mutation (SHP2^E76K/R362E/K364E^). (**E** and **F**) Representative IF staining images (**E**) and statistical analysis (**F**) of SHP2 droplets in SHP2^E76K^ and SHP2^E76K/R362E/K364E^ MSCs (*n* = 6 per group). Data are represented as the mean ± SD. ****P < 0.0001 (2-tailed unpaired *t* test). Scale bar, 15 μm. (**G**) Immunoprecipitation of SHP2 in SHP2^E76K^ and SHP2^E76K/R362E/K364E^ MSCs (*n* = 5 per group) and analyzed using immunoblot analysis with the indicated antibodies. (**H**) Statistical analysis of complex I and complex III activity in SHP2^E76K^ MSCs with and without an additional R362E/K364E mutation. Data are represented as the mean ± SD. **P < 0.01, ***P < 0.001 (2-tailed unpaired *t* test). (**I** and **J**) Statistical analysis of complex I (**I**) and complex III (**J**) activity in WT and SHP2^E76K^ HEK293T cells following the R362E/K364E mutation (*n* = 3 or 5 per group). Data are represented as the mean ± SD. **P < 0.01, ***P < 0.001 (2-way ANOVA with multiple-comparison test). (**K**–**N**) Statistical analysis of soft agar colony formation (**K**), microsphere formation (**L**), percentage of CD184^+^ cells (M), and sarcoma weights (**N**) in SHP2^E76K^ and SHP2^E76K/R362E/K364E^ MSCs. Data are represented as the mean ± SD. **P < 0.01, ****P < 0.0001 (2-tailed unpaired *t* test).

In light of our above findings of mitochondrial complex I and III hyperactivation in the SHP2^E76K^ background, we next sought to clarify the mechanism by which this GOF SHP2 mutation adversely affected complex I and III function. NDUFB8 (an accessory unit of complex I) and UQCRC2 (a component of complex III) are essential subunits for mitochondrial complex I and III function (52, 53). SHP2 was also distributed in the mitochondria of WT and SHP2^E76K^ MSCs (Supplemental Figure 11D). Therefore, we next examined the potential interaction between SHP2 and NDUFB8 and QUCRC2. Co-IP assays showed that SHP2 interacted with NDUFB8 and UQCRC2 in WT and SHP2^E76K^ MSCs (Figure 6A). Moreover, compared with WT MSCs, SHP2^E76K^ MSCs showed markedly decreased binding of SHP2 with NDUFB8 and UQCRC2 (Figure 6A). SHP2 is known as a tyrosine phosphatase, and both complexes I and III occupy several tyrosine-phosphorylated sites (Supplemental Figure 13, A and B). Thus, the disrupted interaction between SHP2 and complexes I and III could reduce the dephosphorylation of complexes I and III but possibly in turn promote their activation in SHP2^E76K^ MSCs.

To investigate how GOF-mutant SHP2 affects its interaction with complexes I and III and to rule out the possibility that mitochondrial SHP2 expression in different genetic backgrounds led to this decreased binding, we compared the expression of SHP2 in mitochondria between WT and SHP2^E76K^ MSCs, and no difference was observed (Supplemental Figure 11D).

LLPS of proteins, also called biomolecule condensation, can dynamically compartmentalize and reintegrate with different proteins to promote oncogenic signaling activation and consequent oncogenesis (31). Given that SHP2^E76K^ can recruit WT SHP2 and undergo LLPS in MSCs (34), we speculated that SHP2 LLPS led to its dissociation from complexes I and III. Supporting this notion, we performed IF staining to measure the level of SHP2 LLPS in WT and SHP2^E76K^ MSCs and found that SHP2 puncta (an indicator of SHP2 LLPS) occurred in SHP2^E76K^ MSCs (Figure 6, B and C). Next, we examined whether inhibition of SHP2 LLPS restores the impaired interaction between SHP2 and complexes I and III in MSCs. We established a transgenic mouse that harbored the SHP2^E76K/R362E/K364E^ mutation (Figure 6D); this triple SHP2 mutation completely abolished SHP2 LLPS without perturbing its overall structure or tyrosine phosphatase activity (34). Bone marrow MSCs were isolated from SHP2^E76K/R362E/K364E^ mice, and SHP2^E76K^ MSCs were considered a control. IF staining verified that SHP2^E76K/R362E/K364E^ MSCs showed a significant decrease in SHP2 puncta formation compared with SHP2^E76K^ MSCs (Figure 6, E and F). We then performed a co-IP assay to assess the interaction between SHP2 and complexes I and III in the mitochondria of SHP2^E76K^ and SHP2^E76K/R362E/K364E^ MSCs. As expected, higher levels of complexes I and III from SHP2-binding proteins were expressed by SHP2^E76K/R362E/K364E^ MSCs than by SHP2^E76K^ MSCs (Figure 6G), suggesting that SHP2 LLPS promotes the separation of SHP2 and complexes I and III in SHP2^E76K^ MSCs.

To further characterize the role of SHP2 LLPS in the malignant transformation of SHP2^E76K^ MSCs, we again isolated MSCs from SHP2^E76K^ and SHP2^E76K/R362E/K364E^ mice. Considering that hyperactive complexes I and III determine the malignant transformation of SHP2 MSCs as mentioned above, we first checked the activity of complexes I and III in SHP2^E76K^ and SHP2^E76K/R362E/K364E^ MSCs. SHP2^E76K/R362E/K364E^ MSCs showed significant inhibition of complex I and III activity compared with SHP2^E76K^ MSCs (Figure 6H). We also transfected HEK293T cells with SHP2^WT^, SHP2^E76K^, SHP2^R362E/K364E^, or SHP2^E76K/R362E/K364E^ to verify whether LLPS-defective mutation inhibited complex I and III activation (Supplemental Figure 14A). The activity of complexes I and III of SHP2^E76K^ HEK293T cells was significantly higher than that of WT HEK293T cells, similar to the phenotype of WT and SHP2^E76K^ MSCs. Additionally, the SHP2^E76K/R362E/K364E^ mutation significantly decreased complex I and III activity compared with the SHP2^E76K^ mutation in HEK293T cells (Figure 6, I and J).

We then asked whether inhibition of SHP2 LLPS prevents malignant transformation of MSCs expressing SHP2^E76K^. The nonanchored colony number (Figure 6K and Supplemental Figure 14B), spheroid number (Figure 6L and Supplemental Figure 14C), and CD184^+^ cells (Figure 6M and Supplemental Figure 14D) of SHP2^E76K/R362E/K364E^ MSCs were significantly lower than those of SHP2^E76K^ MSCs. Moreover, unlike SHP2^E76K^ MSCs, MSCs expressing SHP2^E76K/R362E/K364E^ did not undergo malignant transformation (Figure 6N and Supplemental Figure 14E). Taken together, these data revealed that SHP2 LLPS promotes the malignant transformation of SHP2^E76K^ MSCs by activating complexes I and III.

### Two allosteric inhibitors of SHP2 prevent LLPS, hyperactive complexes I and III, and malignant transformation of SHP2^E76K^ MSCs.

**Figure 7:**
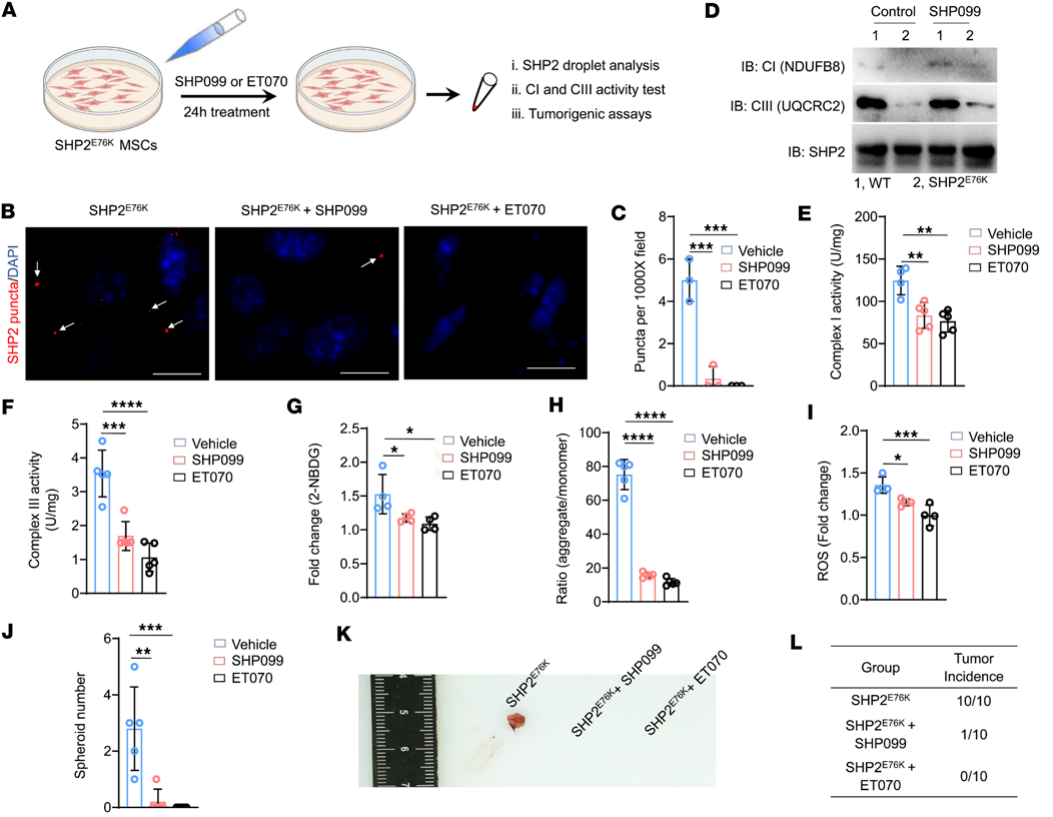
Two allosteric inhibitors of SHP2 (SHP099 and ET070) prevent LLPS, hyperactive energy metabolism, and malignant transformation of SHP2^E76K^ MSCs. (**A**) Schematic diagram depicting the effect of SHP2 inhibitors on mitochondrial complexes I and III and malignant transformation in SHP2^E76K^ MSCs. (**B** and **C**) Representative IF images (**B**) and statistical analysis (**C**) of SHP2 droplets in SHP2^E76K^ MSCs treated with SHP099 and ET070 (*n* = 3 per group). Data are represented as the mean ± SD. ***P < 0.001 (1-way ANOVA with multiple-comparison test). (**D**) Immunoprecipitation of SHP2 in WT and SHP2^E76K^ MSCs with or without SHP099 treatment and analyzed using immunoblot analysis with the indicated antibodies. (**E** and **F**) Statistical analysis of complex I (**E**) and complex III activity (**F**) in WT and SHP2^E76K^ MSCs (*n* = 4 or 5 per group) treated with SHP099 and ET070. Data are represented as the mean ± SD. **P < 0.01, ***P < 0.001, ****P < 0.0001 (1-way ANOVA with multiple-comparison test). (**G**–**I**) Statistical analysis of glucose consumption (**G**), MMP (**H**), and ROS (**I**) in SHP2^E76K^ MSCs treated with SHP099 or ET070. Data are represented as the mean ± SD. *P < 0.05, ***P < 0.001, ****P < 0.0001 (1-way ANOVA with multiple-comparison test). (**J**) Statistical analysis of tumor spheroids in SHP2^E76K^ MSCs following SHP099 and ET070 treatment. Data are represented as the mean ± SD. **P < 0.01, ***P < 0.001 (1-way ANOVA with multiple-comparison test). (**K** and **L**) Representative images (**K**) and incidence (**L**) of tumors induced by SHP2^E76K^ MSCs treated with or without SHP2 allosteric inhibitors (i.e., SHP099 or ET070) (*n* = 10 per group).

Given that SHP2 LLPS promotes complex I and III hyperactivation in SHP2^E76K^ MSC malignant transformation, we next investigated whether SHP2 inhibitors could prevent or alleviate LLPS, hyperactive complexes I and III, and malignant transformation of SHP2^E76K^ MSCs. SHP099 is an allosteric inhibitor of SHP2 that stabilizes the protein in its closed or autoinhibited conformation, thus effectively inhibiting phosphatase of GOF-mutant SHP2 (54, 55). We first treated WT and SHP2^E76K^ MSCs with SHP099 (Figure 7A). A PTP assay revealed that the phosphatase activity of SHP2 was significantly inhibited after SHP099 treatment in both WT and SHP2^E76K^ MSCs (Supplemental Figure 15A). Immunoblotting showed that the ERK1/2 and AMPK/S6 signaling pathways, downstream of SHP2, were also inhibited in both WT and SHP2^E76K^ MSCs, further supporting that SHP099 effectively suppressed SHP2 phosphatase activity (Supplemental Figure 15B).

Given that GOF-mutant SHP2 undergoes LLPS and promotes complex I and III hyperactivation as well as malignant transformation of SHP2^E76K^ MSCs, we then examined the effect of SHP099 on SHP2 LLPS in SHP2^E76K^ MSCs. IF staining of SHP2 puncta showed that SHP2 LLPS was attenuated in SHP2^E76K^ MSCs following SHP099 (at a concentration of 20 μM) treatment (Figure 7, B and C). Since the impaired interaction between SHP2 and complexes I and III was caused by SHP2 LLPS, we treated SHP2^E76K^ MSCs with SHP099 and examined the interaction of SHP2 and complexes I and III. Interestingly, the impaired interaction in SHP2^E76K^ MSCs recovered (Figure 7D). We next measured the activity of complexes I and III following SHP099 treatment. SHP099 significantly inhibited the activity of complexes I and III in MSCs expressing SHP2^E76K^ (Figure 7, E and F). These effects were verified by administration of another SHP2 allosteric inhibitor, ET070 (at a concentration of 10 μM), in SHP2^E76K^ MSCs (Figure 7, B–F). Moreover, we noted that a lower concentration of ET070 than SHP099 effectively inhibited the PTP activity and LLPS of SHP2 as well as the activities of complexes I and III, suggesting that ET070 could be a more potent allosteric inhibitor of SHP2 (Figure 7, B–F).

To better understand the effect of SHP2 allosteric inhibitors on the malignant transformation of SHP2^E76K^ MSCs, we again isolated SHP2^E76K^ MSCs but treated the MSCs with SHP099 and ET070, 2 allosteric inhibitors of SHP2. Glucose consumption, MMP, and ROS production were all significantly suppressed in SHP2^E76K^ MSCs (Figure 7, G–I, and Supplemental Figure 15C). As noted above, SHP2^E76K^ MSCs could transform into sarcoma stem-like cells. These 2 SHP2 inhibitors also prevented the formation of tumor spheroids in SHP2^E76K^ MSC culture (Figure 7J). Moreover, SHP2^E76K^ MSCs with and without SHP099 or ET070 treatment were subcutaneously injected into C57BL/6 mice. On the same day, SHP099 or ET070 was used to treat SHP2^E76K^ MSC-bearing C57BL/6 mice for 2 weeks. These mice were sacrificed at 3 weeks after MSC inoculation, and tumors were collected and weighed. Inhibition of SHP2 by SHP099 and ET070 markedly inhibited tumor formation driven by SHP2^E76K^ MSCs (Figure 7, K and L). Additionally, treatment of ET070 for C57BL/6 mice for 2 weeks also significantly inhibited the growth of sarcoma generated by SHP2^E76K^ MSCs without ET070 pretreatment (Supplemental Figure 15, D and E). Taken together, these results demonstrate that SHP2 allosteric inhibitors can prevent SHP2 LLPS and the associated malignant transformation of SHP2^E76K^ MSCs.

## Discussion

GOF-mutant SHP2-associated Noonan syndrome is usually characterized by short stature with a deformed chest, suggesting the potentially impaired function of MSCs (56, 57). However, no research has been conducted to explore the role of SHP2 in MSC fate determination. We established a cellular model in which MSCs expressing SHP2^E76K^ underwent malignant transformation and initiated sarcomagenesis. Using this model, we revealed that activating SHP2 leads to metabolic dysfunction via dysregulation of mitochondrial complexes I and III. Moreover, we identified SHP2 phase separation as a core mechanism of complex I and III dysregulation, which suggests that targeting SHP2 phase separation can serve as a potential therapeutic target in the treatment of SHP2-associated cancers.

Adult MSCs are well established as a cellular lineage prone to sarcomagenesis (13). Several oncogenic mutations, such as loss of *P53*, *cMyc*, and *Cdkn2a/P16* in MSCs, have been shown to result in spontaneous transformation. SHP2 GOF mutations have also been identified in sarcomas (6). However, the pathogenesis of SHP2–driven sarcomagenesis remains unknown. In this study, we explored this possibility by isolating and purifying MSCs to investigate whether and how SHP2 GOF mutations can drive sarcomagenesis. We show that MSCs expressing SHP2^E76K^ undergo obvious malignant transformation. Moreover, SHP2^E76K^ MSCs transformed into sarcoma stem-like cells. This MSC model thus provides an informative framework for discovering the mechanisms underlying sarcoma initiation and development.

Genetic mutations that lead to metabolic reprogramming are central to maintaining cancer cell proliferation and malleability (58). However, the metabolic effects associated with SHP2-driven tumorigenesis remain largely unknown. Mutations in 2 important metabolic enzymes, IDH and SDH, have been identified in sarcomas (59–61). Both of these enzymes are localized in mitochondria, suggesting that mitochondrial function may play a major role in sarcomagenesis. Our proteomics data also suggest that metabolic dysregulation may be a key stimulator of sarcomagenesis. Our study illustrates the acceleration of mitochondrial respiration at both baseline and maximum levels in MSCs harboring SHP2 GOF mutations. Interestingly, maximum glycolytic activity was also higher in SHP2^E76K^ MSCs, although baseline glycolytic function was unaffected. These findings suggest that mitochondrial function is initially dysregulated in malignant transformation, while aberrant activation of glycolysis may be a contributing factor in later cancer progression.

However, the underlying mechanism by which SHP2 GOF mutations can induce hyperactivity of mitochondrial complexes I and III during tumorigenesis remains elusive. Accumulating evidence suggests that aberrant assembly of condensates (i.e., phase separation) is associated with cancer (29, 33). However, it remains unclear how these well-known membrane protein compartments that form through condensation promote tumorigenesis. We previously reported that SHP2 undergoes LLPS through electrostatic interactions, despite its lack of intrinsically disordered regions (34). Here, we propose that SHP2 LLPS may elevate complex I and III activity during tumorigenesis. This condensate formation disrupts the native interaction between SHP2 and complexes I and III, possibly leading to decreased dephosphorylation levels owing to dissociation of the PTP domain of SHP2 from complexes I and III (54, 62, 63). We speculated that the transition of the SHP2 conformation could cause conformational changes in complexes I and III, leading to constitutive exposure of the enzymatic site. Other potent kinases interact with complexes I and III and promote their activities (64, 65). Although SHP2 LLPS indeed promotes the malignant transformation of SHP2^E76K^ MSCs, the phosphatase role of SHP2 is still critical to tumorigenesis. SHP2-activating mutation leads to persistent activation of the RAS/ERK and mTOR pathways, which is important for cancer cell proliferation. We propose that phosphatase activity and LLPS are 2 independent factors contributing to tumorigenesis. Targeting one of these factors could inhibit cancer malignancy.

In conclusion, our study identifies the mechanism by which a mutant form of SHP2 mediates sarcomagenesis. Specifically, a SHP2 GOF mutant activates mitochondrial metabolism, leading to malignant transformation of MSCs. Moreover, LLPS of SHP2 is a determining factor in complex I and III activity and therefore hyperactive mitochondrial metabolism. Genetic modification or chemical allosteric inhibitors of SHP2 block LLPS and subsequent malignant transformation of SHP2^E76K^ MSCs. Collectively, these findings reveal that SHP2 LLPS mediates mitochondrial dysfunction as part of a core mechanism by which SHP2^E76K^ induces tumorigenesis. These findings suggest that SHP2, and especially its LLPS activity, can serve as promising candidate therapeutic targets in the treatment of SHP2-associated cancers.

## Methods

### Sex as a biological variable.

In this study, both female and male individuals were included, and the results are consistent. No distinctions were made based on sex in the analysis or interpretation of the results.

### Animals.

Mice expressing SHP2^E76K-neo^ (*Ptpn11*^E76K-neo/+^) have been previously reported (57) and were provided by Qu Cheng-Kui’s laboratory at Emory University (Atlanta, Georgia, USA). A neo cassette with a stop codon flanked by *loxP* sites was inserted into the second intron of the *Ptpn11* allele and introduced the mutation GAA (E) to AAA (K) at amino acid 76 of the third exon. The Mx1-Cre mice used in this study were purchased from The Jackson Laboratory. For the induction of the *Ptpn11^E76K/+^* mutation in MSCs, *Mx1*-*Cre* *Ptpn11*^E76K-neo/+^ and *Mx1*-*Cre* *Ptpn11^+/+^* mice received *i.p*. injection of 3 doses of pI-pC (20 μg/mouse) every other day over 5 days. Additionally, SHP2^E76K/R362E/K364E^ mice were provided by Guangya Zhu at Lin ’gang Laboratory (Shanghai, China).

### Mouse MSC isolation.

Mouse MSC isolation has been described previously (1). Briefly, *Mx1*-*Cre* *Ptpn11^E76K/+^* and *Mx1*-*Cre* *Ptpn11^+/+^* mice (6–8 weeks old) were sacrificed and rinsed with 75% ethanol for 3 minutes. Then, the bone marrow was collected separately after muscle dissection. Bone marrow cells were seeded in a culture dish and then moved into the cell incubator. MSCs were observed after 3 days of incubation without any disturbance. To further purify MSCs, cells were collected and stained with biotin-conjugated anti-CD45 antibody and anti-biotin microbeads (Miltenyi Biotec). CD45^+^ hematopoietic cells were depleted using MACS separation columns (Miltenyi Biotec). The purity of MSCs (>95%) was further verified according to the CD45^−^CD140α^+^Sca1^+^ phenotypes by multiparameter FACS analyses (Beckman Coulter**)**.

### PTP activity.

MSCs (1 × 10^6^) were incubated in 10 μL ice-cold lysis buffer on ice for 5 minutes and then centrifuged for 10 minutes, at a speed of 5,000*g*, at 4°C, for total protein purification. A/G-agarose beads and primary antibody (Thermo Fisher Scientific) were incubated with MSCs for 2 hours at room temperature. Subsequently, MSCs were repeatedly centrifuged for 15 minutes, at a speed of 5,000*g*, at 4°C, and the supernatants were used for Western blot (WB) analysis. Isolated SHP2 in the supernatants was further used for PTP activity according to the manufacturer’s instructions (Abcam, K829-100).

### Trilineage differentiation assay of MSCs.

For trilineage differentiation, MSCs with or without SHP2^E76K^ were counted and seeded in a 24-well plate with osteogenic medium (high-glucose DMEM supplemented with 10% [vol/vol] FBS, 10^–7^ M [0.1 μM] dexamethasone, 10 mM β-glycerol phosphate, and 50 μM ascorbate-2-phosphate), adipogenic medium (high-glucose DMEM supplemented with 10% [vol/vol] FBS, 10^−6^ M dexamethasone, 0.5 μM IBMX, and 10 ng/mL insulin), or chondrogenic medium (high-glucose DMEM supplemented with 10% [vol/vol] FBS, TGF-β3 [10 ng/mL], and BMP6 [500 ng/mL]). The medium was changed twice per week, and the cells were maintained in culture for 2–4 weeks. Alizarin red staining, Oil Red O staining, and IF staining with antibodies were used to examine the capacity of differentiation.

### WB.

MSCs and sarcoma tissues were lysed with RIPA buffer (Beyotime Biotechnology). Protein concentration was assessed using a Bradford assay (Bio-Rad). Protein samples (20 μg) were resolved on 8% or 10% polyacrylamide gels and were then electrophoretically transferred to PVDF membranes. The membranes were then blocked for 1 hour in a mix containing nonfat milk and 0.1% Tween 20 in PBS. Membranes were incubated with primary antibodies overnight at 4°C. Finally, HRP-conjugated secondary antibodies were used to identify primary antibodies. Specific signals were detected using an enhanced chemiluminescence WB detection system (Bio-Rad) according to the manufacturer’s instructions. Mouse and/or human actin were used as the loading controls. Detailed data for all antibodies in this study are shown in Supplemental Table 1.

### RNA extraction and quantitative PCR analysis.

An RNAiso Plus kit (Takara) was used according to the manufacturer’s instructions for the extraction of total RNA from MSCs. In addition, a PrimeScript RT reagent kit (Takara) was used to synthesize first-strand cDNA. The expression of various genes was quantified by real-time PCR mixture assays (Takara). Mouse β-actin was used as the internal control. All primer sequences and product sizes are listed in Supplemental Table 2.

### Colony formation in soft agar.

MSCs (1 × 10^3^) were seeded in a 6-well plate for 4 weeks of culture. Of note, this plate was preincubated with MSC culture medium (DMEM + 10% FBS + 0.1% penicillin-streptomycin) mixed with a high concentration of agarose (1.2%). After agarose solidification, WT and SHP2^E76K^ MSCs were seeded on the plate. Next, a low concentration of agarose diluted with MSC medium was added. Cells were cultured for 4 weeks in MSC medium. Finally, a microscope (ZEISS) was used to capture images.

### Tumor spheroid formation.

In the 24-well plate, 0.7% low–melting point agarose was added to each well to lay the base, and the gel was solidified for use. After digestion and centrifugation at a speed of 300*g* for 5 minutes at room temperature, WT and SHP2^E76K^ MSCs were resuspended in 1 mL PBS, centrifuged at 500*g* for 3 minutes, and cleaned twice. These MSCs were then suspended in 1 mL culture medium. Then, 2,000 MSCs were seeded in each well and cultured without serum at 37°C and 5% CO_2_. The formation of microspheres was observed under a microscope and photographed every other day. The formation rate of microspheres was calculated.

### Xenograft tumor formation.

The tumorigenic effect of MSCs with or without mutant SHP2 was determined by a xenograft tumor assay. Briefly, 1 × 10^6^ MSCs were subcutaneously injected into nude (T cell–deficient) mice. After 3 to 4 weeks, the mice were sacrificed. Tumor size and weight were measured. Additionally, C57BL/6 mice also received intramuscular or subcutaneous injections of MSCs (1 × 10^6^) with WT or mutated SHP2 to mimic the formation of sarcoma under the supervision of the immune system.

### Lentivirus transfection of target genes in MSCs.

Three lentiviruses with different fluorescence labels, including ZsGreen, SHP2^WT^, and SHP2^E76K^ plasmids were provided by Hanbio Company for construction of human MSCs bearing SHP2^WT^ and SHP2^E76K^ mutation and MSC labeling. Briefly, after verifying the effective MOI value of the lentivirus, mouse MSCs or human umbilical cord MSCs derived from discarded umbilical cords or MSCs expressing SHP2^E76K^ were seeded into a 24-well plate at a density of 2 × 10^4^. After the confluence reached approximately 40%, we added indicated lentivirus. Following 72 hours of culture, MSCs were examined by fluorescence microscopy and then supplemented with puromycin (2 μg/mL) for stable expression of target genes in MSCs. Additionally, MSCs supplemented with PBS were used as a blank control.

### Serial transplantation of SHP2^E76K^ MSCs.

A total of 1 × 10^6^ SHP2^E76K^ MSCs with green fluorescence were subcutaneously seeded into C57BL/6 mice. After 3 weeks, the mice were sacrificed, and the tumors were collected and digested into single cells with collagen II. A total of 1 × 10^6^ FITC^+^ MSCs were collected and subsequently inoculated into C57BL/6 mice. The process mentioned above was again pursued. Finally, we compared the tumor size and weight and CD184-positive cells.

### Proteomic analysis.

An Orbitrap Fusion mass spectrometer (Thermo Fisher Scientific) was used for proteomic analysis of MSCs expressing SHP2^WT^ and SHP2^E76K^. The ion transfer tube temperature was 320°C, and positive-ion spray voltage was 2.0 kV. The Orbitrap Fusion Lumos was set to the OT-IT mode.

### Seahorse metabolic analysis.

The metabolic pattern of MSCs with WT or GOF-mutant SHP2 was examined by a Seahorse XF analyzer (Agilent). A Seahorse XF Cell Mito Stress Test Kit (103015-100, Seahorse Bioscience) and Glycolysis Kit (103020-100, Seahorse Bioscience) were used to examine mitochondrial respiration and glycolytic function of MSCs according to the manufacturer’s instructions.

### ATP production assay.

The ATP content of MSCs expressing SHP2^WT^ and SHP2^E76K^ was examined by an ATP kit (Beyotime, S0026). Briefly, the culture medium was removed, and the cells were lysed by adding 200 μL of lysate to each well of the 6-well plate. Centrifugation was performed at 12,000*g* at 4°C for 5 minutes after lysis, and the supernatant was collected for subsequent determination. Three hundred microliters of the supernatant from MSCs was used to detect ATP. ATP assay mix was added to MSCs. ATP-driven chemiluminescence signals were recorded with a luminescence microplate reader.

### Glucose consumption.

MSCs with or without the SHP2^E76K^ mutation were incubated with 2-NBDG (100 μM) in a 37°C water bath for 2 hours. Next, MSCs were washed and centrifuged at a speed of 300*g* for 5 minutes at room temperature. Finally, flow cytometry was used to analyze the population of 2-NBDG^+^ MSCs and the mean value of FITC fluorescence.

### Glutamate concentration test.

The amount of glutamate in MSCs was quantified by a glutamate colorimetric assay kit (K629-100, Abcam). Briefly, MSCs were homogenized in assay buffer and then centrifuged at 13,000*g* for 10 minutes at 4°C. The supernatant was mixed with glutamate enzyme mix for quantification of glutamate content.

### C^13^-labeled glutamine tracing assay.

For glutamine metabolomics, WT and SHP2^E76K^ MSCs were seeded at 10^6^ cells per 10 cm plate and cultured in a glutamine-supplemented DMEM supplemented with 10% FBS for 6 hours. Next, cells were washed and incubated in a glutamine-free DMEM supplemented with 10% FBS and 4 mM l-glutamine, (98% atom ^13^C, 95% (chemically pure), MilliporeSigma 605166) or glutamine-supplemented DMEM complete medium (8 mL) under 5% O_2_ for 8 hours. Subsequently, cells were washed with ice-cold PBS, lysed with 400 μL methanol, and quickly scraped and stored in liquid nitrogen. The samples were processed by 5 cycles of 1-minute ultrasonication and 1-minute interval in ice-water bath and stood for 30 minutes at –20°C. After centrifugation at 15,000 RCF for 15 minutes at 4°C, a 100 μL supernatant was evaporated to dryness under nitrogen stream. The residue was reconstituted in 30 μL of 20 mg/mL methoxyamine hydrochloride in pyridine, and the resulting mixture was incubated at 37°C for 90 minutes. A total of 30 μL of MTBSTFA (with 1% TBDMCS) was added into the mixture and derivatized at 55°C for 60 minutes prior to gas chromatography–MS metabolomics analysis.

### BCAA test.

The BCAA content was examined by a Branched Chain Amino Acid Colorimetric Assay Kit (K564-100, Abcam). In brief, MSCs were homogenized in assay buffer and then centrifuged at 15,000*g* for 10 minutes at 4°C. The supernatant was mixed with enzyme mix for quantification of BCAA content.

### MMP test.

MSCs expressing SHP2^WT^ and SHP2^E76K^ were harvested to determine the MMP using a JC-1 assay kit (MedChemExpress, HY-K0601). Briefly, MSCs (1 × 10^5^) were suspended in JC-1 staining buffer and cultured in an incubator at 37°C for 20 minutes. After incubation, MSCs were washed 3 times with JC-1 washing buffer. Flow cytometry was used to analyze the MMP in MSCs.

### Lactate production assay.

The production of lactate in MSCs was examined by a lactate colorimetric/fluorometric assay kit (Abcam K607-100). Briefly, MSCs were subcultured into a 96-well plate. Lactate assay buffer and lactate enzyme mix were both added to the 96-well plate. After incubation at room temperature for 30 minutes, the OD value at 570 nm detected via a microplate reader was considered the relative content of lactate.

### Mitochondrial complex activity assay.

The mitochondrial complex activity of MSCs and HEK293T cells (a gift from Guangya Zhu) was examined by Mitochondrial Complexes Kits (BC0515, BC3230, BC3240, BC0945, and BC1445, Solarbio) according to the manufacturer’s instructions. Briefly, MSCs were homogenized and centrifuged at 600*g* at 4°C for 10 minutes. The supernatant was then secondarily centrifuged at 11,000*g* at 4°C for 15 minutes. The sediment was ultrasonicated, and the activity was measured via an ultraviolet spectrophotometer (Thermo Fisher Scientific).

### ROS test.

MSCs with or without SHP2 GOF mutations were collected for ROS testing according to the manufacturer’s instructions. Briefly, MSCs were collected and incubated with DCFH-DA or MitoSOX Red diluted in DMEM (1:1,000) for 20 minutes at 37°C. After adding the fluorescence probe, the fluorescence was examined by flow cytometry (all from BD Biosciences).

### IF and immunohistochemistry.

Immunostaining for different markers was performed as previously described (46). Briefly, sections were prefixed with 4% paraformaldehyde in PBS. Nonspecific binding was blocked by incubation for 1 hour at room temperature with 10% normal serum diluted in 1% bovine serum albumin (BSA; Jackson ImmunoResearch Laboratories) and 0.25% Triton X-100 (MilliporeSigma). The sections were then incubated with primary antibodies diluted with 1% BSA + 0.25% Triton X-100 at 4°C overnight. After washing, the sections were incubated with the appropriate secondary antibodies (Alexa Fluor 488– and Alexa Fluor 594–conjugated antibodies that were diluted with 1% BSA + 0.25% Triton X-100) in the dark at room temperature for 2 hours; counterstaining was performed with 4,6-diamidino-2-phenylindole (1:5,000). All fluorescently stained images were captured using a ZEISS Axio Observer. Signals from all channels were collected individually and overlaid in Adobe Photoshop.

### Treatment with small molecular inhibitors.

WT and SHP2^E76K^ MSCs were treated with or without metformin (1 mM), atovaquone (10 μM), SHP099 (20 μM), and ET070 (10 μM) for 24 hours. C57BL/6 mice with or without subcutaneous injection of SHP2^E76K^ MSCs were treated with metformin (50 mg/kg), atovaquone (50 mg/kg), SHP099 (30 mg/kg), and ET070 (10 mg/kg) through *i.p*. injection every other day for 2 weeks.

### Statistics.

All experiments were repeated at least 3 times. Data are reported as the mean ± SD. Statistical analyses between 2 groups were performed using unpaired 2-tailed *t* tests. When assessing multiple groups, 1- or 2-way ANOVA was used with Tukey’s multiple-comparison test. All statistical analyses were performed using Prism 8 (GraphPad). *P* < 0.05 was considered statistically significant in this study.

### Study approval.

All the mouse experiments of this study were approved by the Animal Care and Use Committees at Anhui Medical University (Protocol: LLSC20210972). Additionally, the experiments on umbilical cord MSCs were approved by the Biomedical Ethics Committee of Anhui Medical University (Protocol: 20200821 and 20210594).

### Data availability.

The mass spectrometry proteomics data have been deposited to the ProteomeXchange Consortium (http://proteomecentral.proteomexchange.org) via the iProX partner repository (66, 67), with the data set identifier PXD040487. All data needed to evaluate the conclusions in the paper are presented in the paper and/or the supplement (including Supporting Data Values).

## Author contributions

CK, ZT, LL, BL, LZ, JZ, JL, MW, XL, KL, YL, and FY were responsible for collection and assembly of data; CK, SW, and HZ were responsible for data analysis and manuscript writing; and HZ and SW were responsible for conception and design, data analysis and interpretation, and manuscript revision and final approval of the manuscript. In this study, CK led all the experiments and completed the majority of them and therefore should be listed first. ZT completed some of the significant experiments and should be listed second. LL assisted CK in several experiments and should be listed third.

## Supplementary Material

Supplemental data

Unedited blot and gel images

Supporting data values

## Version 1 03/07/2024

In-Press Preview

## Version 2 04/22/2024

Electronic publication

## References

[1] Dong L (2016). Leukaemogenic effects of Ptpn11 activating mutations in the stem cell microenvironment. Nature.

[2] Zheng H (2013). Induction of a tumor-associated activating mutation in protein tyrosine phosphatase Ptpn11 (Shp2) enhances mitochondrial metabolism, leading to oxidative stress and senescence. J Biol Chem.

[3] Mossmann D (2018). mTOR signalling and cellular metabolism are mutual determinants in cancer. Nat Rev Cancer.

[4] Kan C (2018). SHP2-mediated signal networks in stem cell homeostasis and dysfunction. Stem Cells Int.

[5] Tartaglia M (2006). Diversity and functional consequences of germline and somatic PTPN11 mutations in human disease. Am J Hum Genet.

[6] Shukla N (2012). Oncogene mutation profiling of pediatric solid tumors reveals significant subsets of embryonal rhabdomyosarcoma and neuroblastoma with mutated genes in growth signaling pathways. Clin Cancer Res.

[7] Soliman H (2021). Multipotent stromal cells: one name, multiple identities. Cell Stem Cell.

[8] Kaplan FS (2020). Fibrodysplasia ossificans progressiva (FOP): a disorder of osteochondrogenesis. Bone.

[9] Cong Q (2021). A self-amplifying loop of YAP and SHH drives formation and expansion of heterotopic ossification. Sci Transl Med.

[10] Guarnerio J (2015). A genetic platform to model sarcomagenesis from primary adult mesenchymal stem cells. Cancer Discov.

[11] Kan C (2019). BMP-dependent, injury-induced stem cell niche as a mechanism of heterotopic ossification. Stem Cell Res Ther.

[12] Lonetto G (2019). Mutant p53-dependent mitochondrial metabolic alterations in a mesenchymal stem cell-based model of progressive malignancy. Cell Death Differ.

[13] Rodriguez R (2012). Modeling sarcomagenesis using multipotent mesenchymal stem cells. Cell Res.

[14] Shetzer Y (2014). The onset of p53 loss of heterozygosity is differentially induced in various stem cell types and may involve the loss of either allele. Cell Death Differ.

[15] Rubio R (2013). The differentiation stage of p53-Rb-deficient bone marrow mesenchymal stem cells imposes the phenotype of in vivo sarcoma development. Oncogene.

[16] Teng IW (2011). Targeted methylation of two tumor suppressor genes is sufficient to transform mesenchymal stem cells into cancer stem/initiating cells. Cancer Res.

[17] Yang WT (2013). Ptpn11 deletion in a novel progenitor causes metachondromatosis by inducing hedgehog signalling. Nature.

[18] Vander Heiden MG, DeBerardinis RJ (2017). Understanding the intersections between metabolism and cancer biology. Cell.

[19] Kim J, DeBerardinis RJ (2019). Mechanisms and implications of metabolic heterogeneity in cancer. Cell Metab.

[20] Chen H, Chan DC (2017). Mitochondrial dynamics in regulating the unique phenotypes of cancer and stem cells. Cell Metab.

[21] Altieri DC (2022). Mitochondria in cancer: clean windmills or stressed tinkerers?. Trends Cell Biol.

[22] Ashton TM (2018). Oxidative phosphorylation as an emerging target in cancer therapy. Clin Cancer Res.

[23] Hayes J (2020). Oxidative stress in cancer. Cancer Cell.

[24] Muller FL (2004). Complex III releases superoxide to both sides of the inner mitochondrial membrane. J Biol Chem.

[25] Hirst J (2013). Mitochondrial complex I. Annu Rev Biochem.

[26] Sabharwal SS, Schumacker PT (2014). Mitochondrial ROS in cancer: initiators, amplifiers or an Achilles’ heel?. Nat Rev Cancer.

[27] Zhu GG (2020). Genomic profiling identifies association of *IDH1/IDH2* mutation with longer relapse-free and metastasis-free survival in high-grade chondrosarcoma. Clin Cancer Res.

[28] Weinberg F (2010). Mitochondrial metabolism and ROS generation are essential for Kras-mediated tumorigenicity. Proc Natl Acad Sci U S A.

[29] Jiang S (2020). Protein phase separation and its role in tumorigenesis. Elife.

[30] Alberti S, Dormann D (2019). Liquid-liquid phase separation in disease. Annu Rev Genet.

[31] Guo C (2020). ENL initiates multivalent phase separation of the super elongation complex (SEC) in controlling rapid transcriptional activation. Sci Adv.

[32] Mehta S, Zhang J (2022). Liquid-liquid phase separation drives cellular function and dysfunction in cancer. Nat Rev Cancer.

[33] Liu QX (2021). Glycogen accumulation and phase separation drives liver tumor initiation. Cell.

[34] Zhu GY (2020). Phase separation of disease-associated SHP2 mutants underlies MAPK hyperactivation. Cell.

[35] Park D (2012). Endogenous bone marrow MSCs are dynamic, fate-restricted participants in bone maintenance and regeneration. Cell Stem Cell.

[36] Kramann R (2015). Perivascular Gli1+ progenitors are key contributors to injury-induced organ fibrosis. Cell Stem Cell.

[37] Houlihan DD (2012). Isolation of mouse mesenchymal stem cells on the basis of expression of Sca-1 and PDGFR-α. Nat Protoc.

[38] Chen YY (2006). Mutations of the PTPN11 and RAS genes in rhabdomyosarcoma and pediatric hematological malignancies. Genes Chromosomes Cancer.

[39] Singh S (2022). Targeting KDM4 for treating PAX3-FOXO1-driven alveolar rhabdomyosarcoma. Sci Transl Med.

[40] Zibat A (2010). Activation of the hedgehog pathway confers a poor prognosis in embryonal and fusion gene-negative alveolar rhabdomyosarcoma. Oncogene.

[41] Rosland GV (2009). Long-term cultures of bone marrow-derived human mesenchymal stem cells frequently undergo spontaneous malignant transformation. Cancer Res.

[42] Reya T (2001). Stem cells, cancer, and cancer stem cells. Nature.

[43] Balani S (2017). Modeling the process of human tumorigenesis. Nat Commun.

[44] Cornaz-Buros S (2014). Targeting cancer stem-like cells as an approach to defeating cellular heterogeneity in Ewing sarcoma. Cancer Res.

[45] Genadry KC (2018). Soft tissue sarcoma cancer stem cells: an overview. Front Oncol.

[46] Kan C (2018). Gli1-labeled adult mesenchymal stem/progenitor cells and hedgehog signaling contribute to endochondral heterotopic ossification. Bone.

[47] McGuirk S (2020). Metabolic fitness and plasticity in cancer progression. Trends Cancer.

[48] Pfanner N (2019). Mitochondrial proteins: from biogenesis to functional networks. Nat Rev Mol Cell Biol.

[49] Martínez-Reyes I, Chandel N (2020). Mitochondrial TCA cycle metabolites control physiology and disease. Nat Commun.

[50] Sun C (2017). MitoQ regulates autophagy by inducing a pseudo-mitochondrial membrane potential. Autophagy.

[51] Zhang MS (2013). RAS and ROS in rhabdomyosarcoma. Cancer Cell.

[52] Piekutowska-Abramczuk D (2018). NDUFB8 mutations cause mitochondrial complex I deficiency in individuals with Leigh-like encephalomyopathy. Am J Hum Genet.

[53] Miyake N (2013). Mitochondrial complex III deficiency caused by a homozygous UQCRC2 mutation presenting with neonatal-onset recurrent metabolic decompensation. Hum Mutat.

[54] Chen YNP (2016). Allosteric inhibition of SHP2 phosphatase inhibits cancers driven by receptor tyrosine kinases. Nature.

[55] LaRochelle JR (2018). Structural reorganization of SHP2 by oncogenic mutations and implications for oncoprotein resistance to allosteric inhibition. Nat Commun.

[56] Araki T (2004). Mouse model of Noonan syndrome reveals cell type- and gene dosage-dependent effects of Ptpn11 mutation. Nat Med.

[57] Xu D (2011). Non-lineage/stage-restricted effects of a gain-of-function mutation in tyrosine phosphatase Ptpn11 (Shp2) on malignant transformation of hematopoietic cells. J Exp Med.

[58] Tomasetti C (2017). Stem cell divisions, somatic mutations, cancer etiology, and cancer prevention. Science.

[59] Li L (2017). Metabolic enzymes in sarcomagenesis: progress toward biology and therapy. BioDrugs.

[60] Suijker J (2015). The oncometabolite D-2-hydroxyglutarate induced by mutant IDH1 or -2 blocks osteoblast differentiation in vitro and in vivo. Oncotarget.

[61] Selak MA (2005). Succinate links TCA cycle dysfunction to oncogenesis by inhibiting HIF-alpha prolyl hydroxylase. Cancer Cell.

[62] Hernansanz-Agustín P (2017). Mitochondrial complex I deactivation is related to superoxide production in acute hypoxia. Redox Biol.

[63] Letts J (2019). Structures of respiratory supercomplex I+III_2_ reveal functional and conformational crosstalk. Mol Cell.

[64] Hebert-Chatelain E (2012). Preservation of NADH ubiquinone-oxidoreductase activity by Src kinase-mediated phosphorylation of NDUFB10. Biochim Biophys Acta.

[65] Schilling B (2005). Mass spectrometric identification of a novel phosphorylation site in subunit NDUFA10 of bovine mitochondrial complex I. FEBS Lett.

[66] Ma J (2019). iProX: an integrated proteome resource. Nucleic Acids Res.

[67] Chen T (2022). iProX in 2021: connecting proteomics data sharing with big data. Nucleic Acids Res.

