# Supplementary material for: Phase separation of SHP2E76K promotes malignant transformation of mesenchymal stem cells by activating mitochondrial complexes
Source: JCI Insight. 2024 Mar 7;9(8):e170340. doi: 10.1172/jci.insight.170340 (PMC11141883; doi:10.1172/jci.insight.170340)
Supplement: Supplemental data [file jciinsight-9-170340-s008.pdf]

# Supplementary information

## Supplementary Table 1-2

### Supplementary Table 1, Summary of antibodies used in this study

| ID | Company | Cat | Clone number |
|---|---|---|---|
| SHP2 | CST | 3397S | D50F2 |
| ERK | CST | 4695 | 137F5 |
| p-ERK | CST | 4370 | D13.14.4E |
| Vimentin | Abcam | Ab193555 | EPR3776 |
| α-SMA | Abcam | Ab32575 | E184 |
| Desmin | Abcam | Ab15200 | Polyclonal |
| Ki-67 | Abcam | Ab15580 | Polyclonal |
| p-AKT | CST | 4060 | D9E |
| AKT | CST | 2920 | 40D4 |
| p-mTOR | Invitrogen | SB233656A | Polyclonal |
| mTOR | Invitrogen | PA5-34663 | Polyclonal |
| HIF1α | Invitrogen | MA1-16504 | H1alpha67 |
| β-Actin | CST | 3700 | 8H10D10 |
| β-Tubulin | Invitrogen | 32-2600 | 2 28 33 |
| HSP60 | Invitrogen | PA5-34760 | Polyclonal |
| OXPHOS Rodent WB Antibody Cocktail | Thermo | 45-8099 | Cocktail |
| NDUFB8 | Abcam | Ab251160 | EPR15961 |
| UQCRC2 | Abcam | Ab240368 | EPR13051 |
| AMPK | CST | 5831 | D5A2 |
| p-AMPK | CST | 50081 | D4D6D |
| S6 | CST | 2217 | 5G10 |
| p-S6 | CST | 4858 | D57.2.2E |
| ACC | CST | 3662 | Polyclonal |
| p-ACC | CST | 3661 | Ser79 |
| IBA1 | CST | 17198 | E4O4W |
| CD133 | Invitrogen | PA5-38014 | Polyclonal |
| CD133-APC | Biolegend | 141208 | 315-2C11 |
| Frizzled-1 Antibody, anti-mouse, PE | Miltenyi Biotec | 130-112-397 | REA603 |
| CD271- Alexa Fluor™ 488 | eBioscience | 53-9400-42 | ME20.4 |
| CD184-PE | Biolegend | 146505 | L276F12 |
| S100A4 | Abcam | Ab218512 | S100A4/1482 |
| MyoD1 | CST | 13812 | D8G3 |

| | | | |
|---|---|---|---|
| Myogenin | Invitrogen | 14-5643-80 | F5D |
| Caldesmon | Thermo | PA5-27719 | Polyclonal |
| S100-β | Abcam | Ab52642 | EP1576Y |
| Sox9 | Thermo | PA5-81966 | Polyclonal |
| Aggrecan | Thermo | MA5-42646 | 6L4T2 |

**Supplementary Table 2, Summary of primers used in this study**

| Primer ID | Sequence (5' to 3') |
|---|---|
| *Neo-F* | TACCTTGAGGTTAGTGAACGTCA |
| *Neo-R* | CGCTCTCGTTTTCCCCATAATC |
| *β-actin-F* | GGCTGTATTCCCCTCCATCG |
| *β-actin-R* | CCAGTTGGTAACAATGCCATGT |
| *18s-F* | TAGAGGGACAAGTGGCGTTC |
| *18s-R* | CGCTGAGCCAGTCAGTGT |
| *CytB-F* | CCACTCATTCATTGACCTACCT |
| *CytB-R* | GCTCCGTTTGCGTGTATATATC |

**Supplemental Figures 1-15**

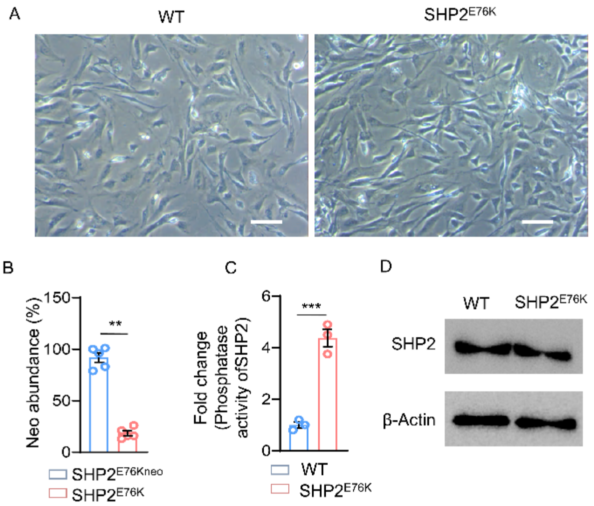


**Supplemental Figure 1. Measurement of Mx1-cre-mediated recombination in WT and SHP2$^{E76K}$ MSCs.** **A** Representative image of WT and SHP2$^{E76K}$ MSCs in culture. **B** Statistical analysis of neo abundance in MSCs expressing SHP2$^{E76K\text{-}neo}$ or SHP2$^{E76K}$ (n=5 per group). Data are represented as the means ± SD. $^{**}p < 0.01$ (two-tailed unpaired t test). **C** Statistical analysis of SHP2 phosphatase activity in WT and SHP2$^{E76K}$ MSCs (n=3 per group). Data are represented as the means ± SD. $^{***}p < 0.001$ (two-tailed unpaired t test). **D** Western blot analysis of SHP2 expression in Mx1-cre and Mx1-cre; SHP2$^{E76K}$ mouse MSCs.

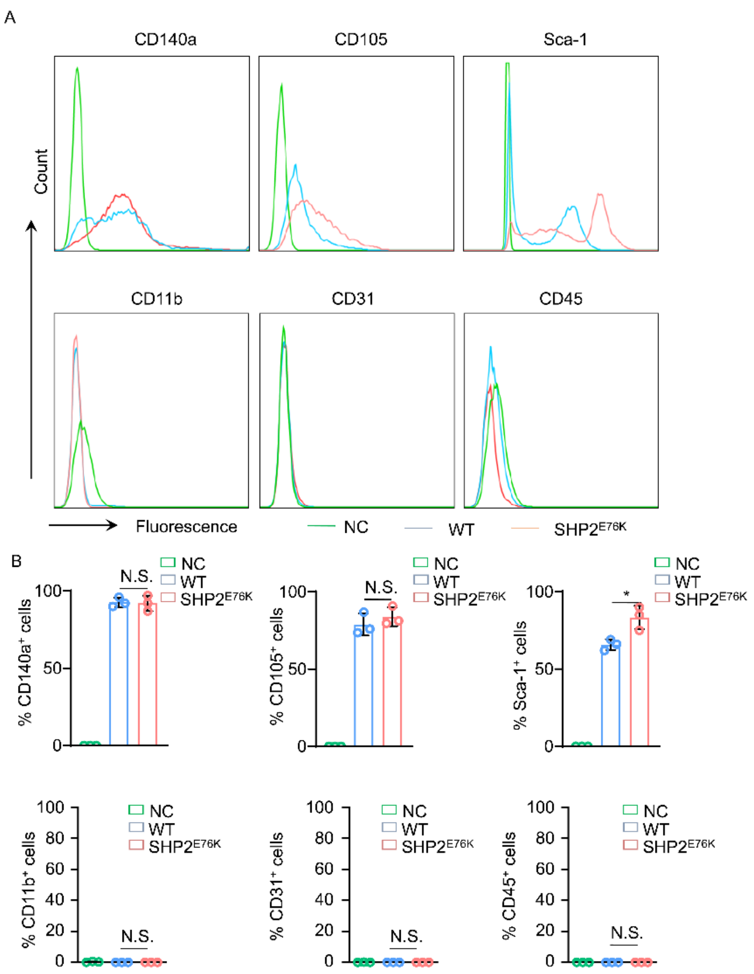


**Supplemental Figure 2. Characterization of WT and SHP2$^{E76K}$ MSCs. A** Flow cytometry validation of MSCs using different markers, including CD140a, CD105, Sca1, CD11b, CD31 and CD45. **B** Statistical analysis of the population of CD140a$^+$, CD105$^+$, Sca1$^+$, CD11b$^+$, CD31$^+$ or CD45$^+$ cells among WT and SHP2$^{E76K}$ MSCs (n=3 per group). Data are represented as the means ± SD. **p* < 0.05, N.S. indicates no significance (two-tailed unpaired t test). indicates no significance (two-tailed unpaired t test).

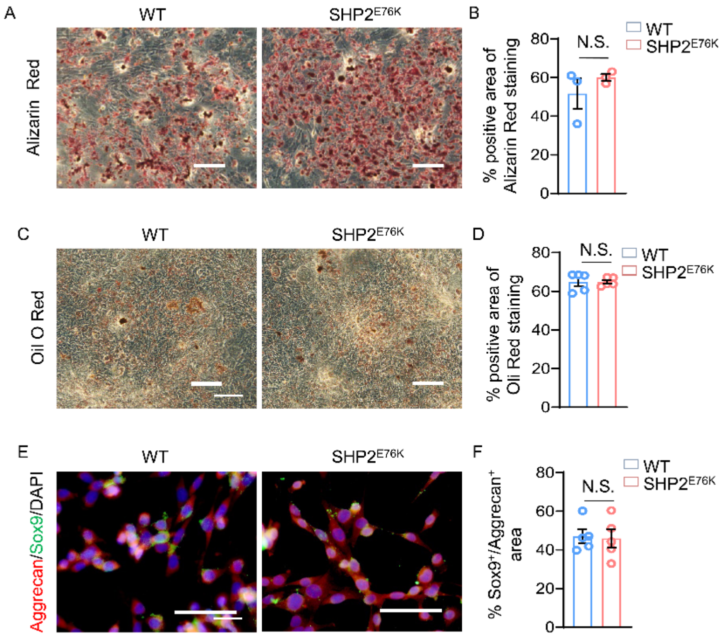


**Supplemental Figure 3. There was no significant difference in trilineage differentiation capacity between WT and SHP2$^{E76K}$ MSCs. A, B** Representative images (**A**) and statistical analysis (**B**) of Alizarin Red staining in MSCs with or without GOF mutant SHP2 (n=3 per group). Data are represented as the means ± SD. N.S. indicates no significance (two-tailed unpaired t test). Scale bar, 200 µm. **C, D** Representative images (**C**) and statistical analysis of Oil Red O staining (**D**) in MSCs with or without GOF mutant SHP2 (n=5 per group). Data are represented as the means ± SD, N.S. indicates no significance (two-tailed unpaired t test). Scale bar, 100 µm. **E, F** Representative IF images (**E**) and statistical analysis (**F**) of the SOX9$^{+}$/Aggrecan$^{+}$ area in MSCs with or without GOF mutant SHP2 (n=5 per group). Data are represented as the means ± SD, N.S. indicates no significance (two-tailed unpaired t test). Scale bar, 200 µm.

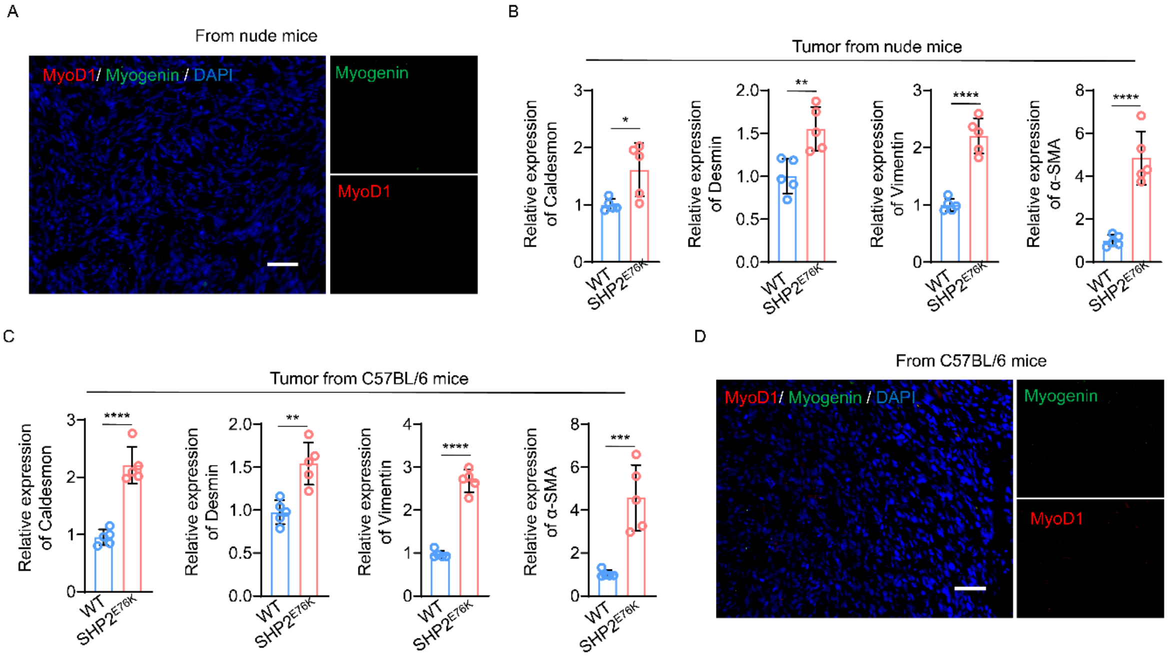


**Supplemental Figure 4. SHP2$^{E76K}$ MSC-initiated sarcomas did not express MyoD1 and Myogenin. A** Representative immunofluorescence images of MyoD1 and Myogenin staining in sarcomas developed from nude mice. Scale bar, 100 µm. **B** Statistical analysis of Caldesmon, Desmin, Vimentin and α-SMA in normal subcutaneous tissue (with WT MSC injection) and sarcomas (with SHP2$^{E76K}$ MSC injection) from nude mice (n=5 per group). Data are represented as the mean ± SD. *$p < 0.05$, **$p < 0.01$, ****$p < 0.0001$ (two–tailed unpaired *t* test). **C** Statistical analysis of Caldesmon, Desmin, Vimentin and α-SMA in normal subcutaneous tissues (with WT MSC injection) and sarcomas (with SHP2$^{E76K}$ MSC injection) from C57BL/6 mice (n=5 per group). Data are represented as the mean ± SD. **$p < 0.01$, ***$p < 0.001$, ****$p < 0.0001$ (two–tailed unpaired *t* test). **D** Representative immunofluorescence images of MyoD1 and Myogenin staining in sarcomas developed from C57BL/6 mice. Scale bar, 100 µm.

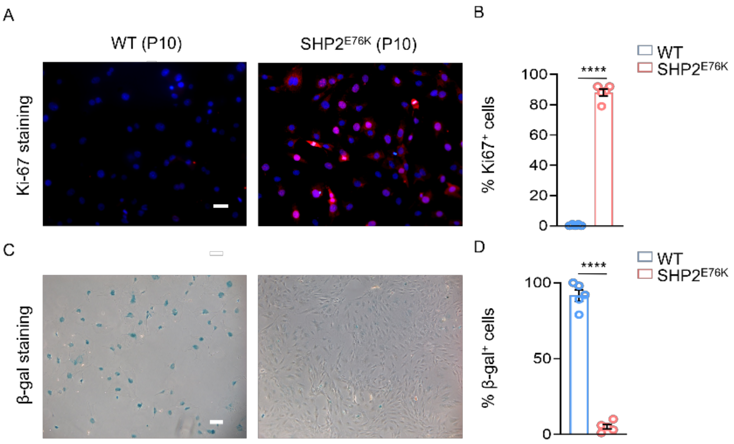


**Supplemental Figure 5. The proliferation capacity of SHP2$^{E76K}$ MSCs gradually accelerated, but WT MSCs progressively became senescent following successive passages. A, B** Representative immunofluorescence images (**A**) and statistical analysis (**B**) of Ki67 staining in WT and mutant MSCs at the indicated passages (n=5 per group). Scale bar, 200 µm. Data are represented as the means ± SD. $****p < 0.00001$ (two-tailed unpaired t test). **C, D** Representative images (**C**) and statistical analysis (**D)** of β-gal staining in WT and mutant MSCs at the indicated passages (n=5 per group). Scale bar, 200 µm. Data are represented as the means ± SD. $****p < 0.00001$ (two-tailed unpaired t test).

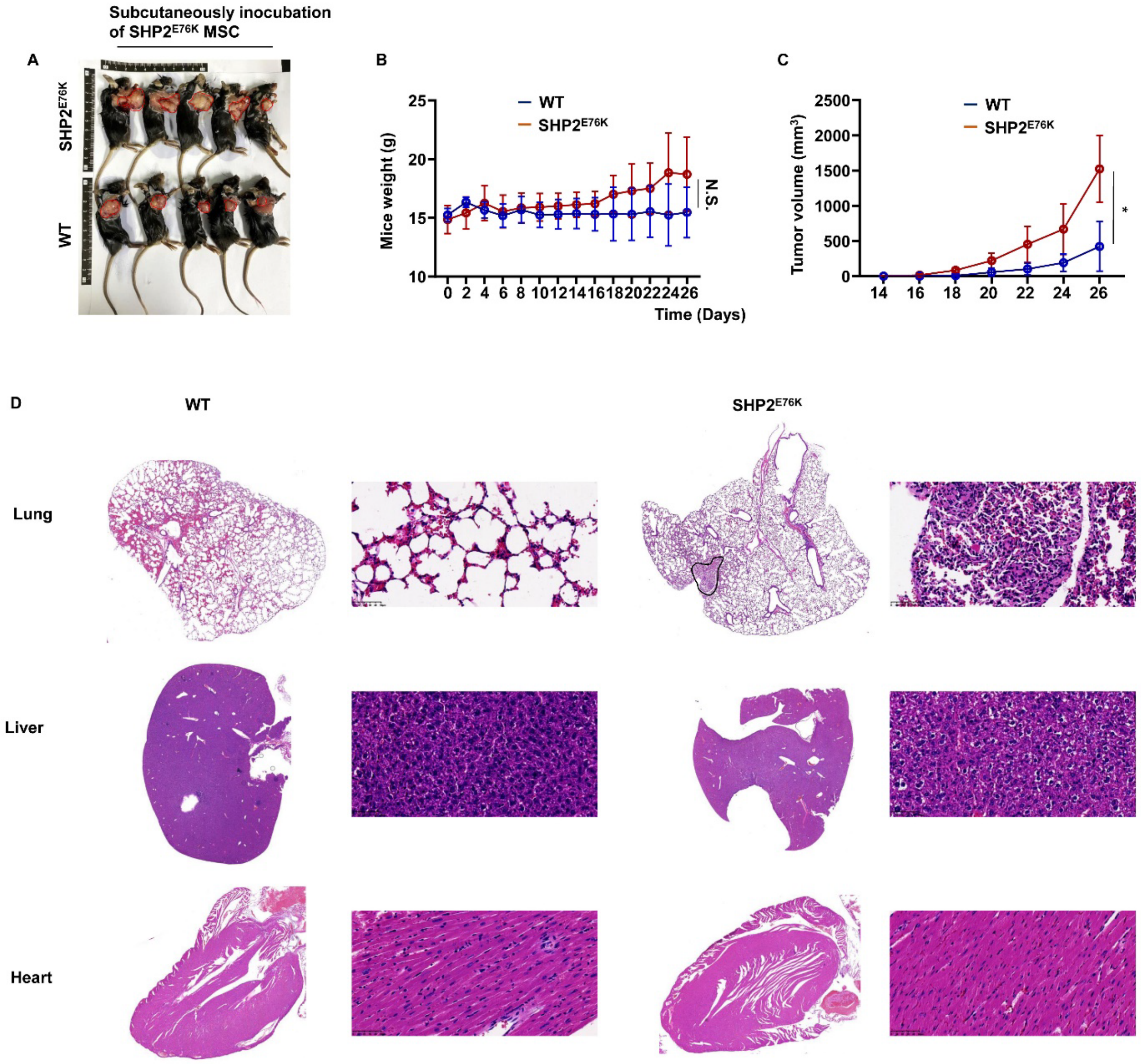


**Supplemental Figure 6. SHP2$^{E76K}$ MSCs can initiate sarcomagenesis and lung metastasis in C57BL/6 mice.** **A** Representative image of tumors in WT and Mx1-cre; SHP2$^{E76K}$ mice with SHP2$^{E76K}$ MSCs inoculation. **B**, **C** Statistical analysis of mice wight (**B**) and tumor volume (**C**) of WT and Mx1-cre; SHP2$^{E76K}$ mice (n=5 per group) with SHP2$^{E76K}$ MSCs inoculation at different time points. Data are represented as the means ± SD. *$p < 0.05$, N.S. indicates no significance (two-tailed unpaired t test). **D** Representative HE staining images of Lung, liver and heart in C57BL/6 mice inoculated with WT or SHP2$^{E76K}$ MSCs. Scale bar, 50 μm.

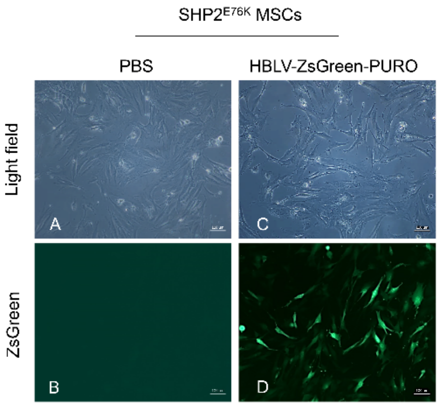


**Supplemental Figure 7. Lentivirus transfection of ZsGreen in SHP2$^{E76K}$ MSCs. A, B** Representative light field images (**A**) and fluorescence images (**B**) of SHP2$^{E76K}$ MSCs without lentivirus transfection. **C, D** Representative light field images (**C**) and fluorescence images (**D**) of SHP2$^{E76K}$ MSCs overexpressing ZsGreen. Scale bar, 100 µm.

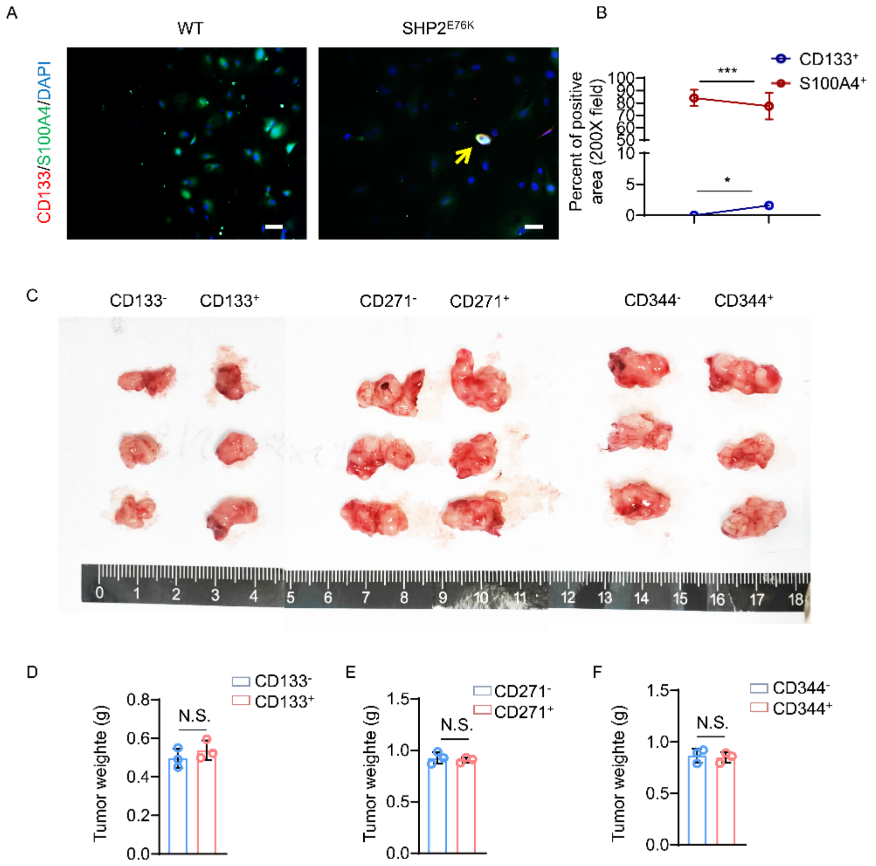


**Supplemental Figure 8 CD133, CD271 and CD344 positive cells did not exhibit difference in tumor progression among SHP2$^{E76K}$ MSCs. A, B** Representative IF staining images (**A**) and statistical analysis (**B**) of S100A4 and CD133 in WT and SHP2$^{E76K}$ MSCs. Scale bar, 200 μm. Data are represented as the mean ± SD. *$p < 0.05$, ***$p < 0.001$ (two–tailed unpaired $t$ test). **C-F** Representative tumor images and statistical analysis of tumor developed by CD133$^-$, CD133$^+$, CD271$^-$, CD271$^+$, CD344$^-$ and CD344$^+$ SHP2$^{E76K}$ MSCs (n=3 per group).

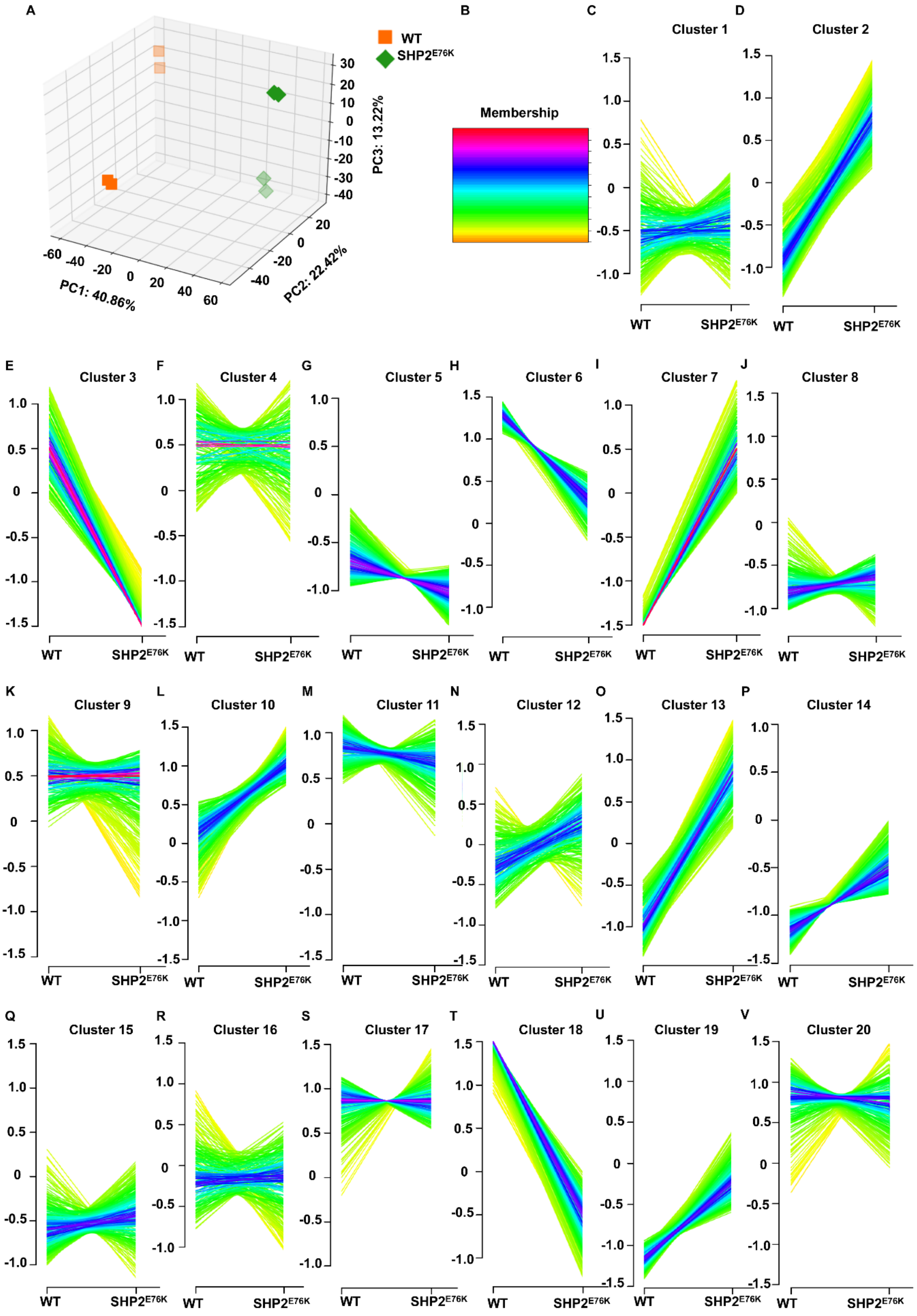


**Supplemental Figure 9 PCA and trend analysis of WT and SHP2$^{E76K}$ MSCs. A** PCA of the WT and SHP2$^{E76K}$ MSCs. **B-V** Trend analysis of WT and SHP2$^{E76K}$ MSCs.

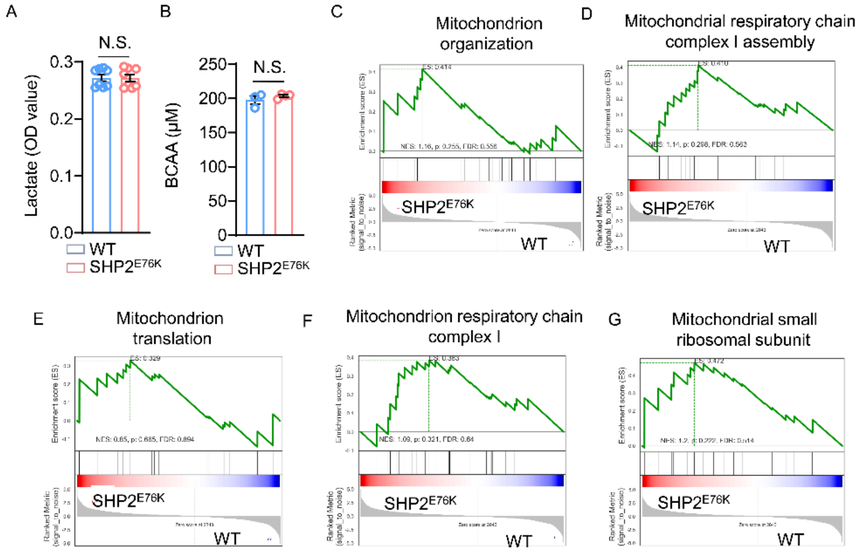


**Supplemental Figure 10. Some metabolic parameters of SHP2$^{E76K}$ MSCs did not change significantly, but mitochondria-associated proteins were enriched in SHP2$^{E76K}$ MSCs. A** Statistical analysis of the content of lactate in WT and SHP2$^{E76K}$ MSCs (n=8 per group). Data are represented as the means ± SD. N.S. indicates no significance (two-tailed unpaired *t* test). **B** Statistical analysis of the content of BCCA in WT and SHP2$^{E76K}$ MSCs (n=3 per group). Data are represented as the means ± SD. N.S. indicates no significance (two-tailed unpaired *t* test). **C-G** GSEA of mitochondria-associated proteins in WT and SHP2$^{E76K}$ MSCs.

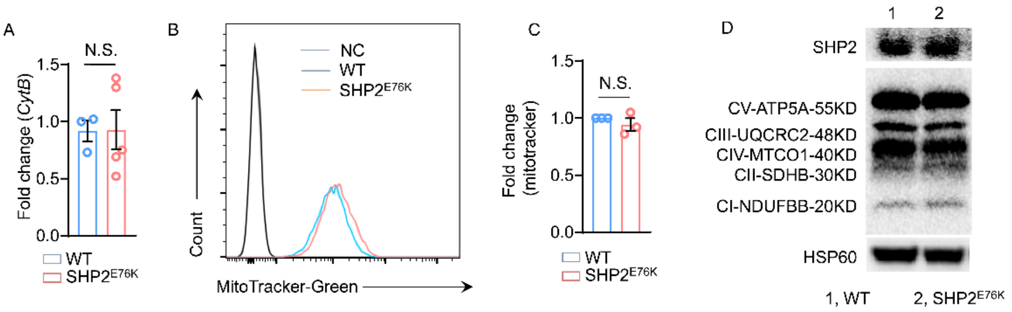


**Supplemental Figure 11. Mitochondrial biogenesis, gene expression and complex expression in SHP2$^{E76K}$ MSCs did not change significantly. A** Statistical analysis of *CytB* expression in WT and SHP2$^{E76K}$ MSCs (n=3 or 5 per group). Data are represented as the means ± SD. N.S. indicates no significance (two-tailed unpaired *t* test). **B, C** Representative image of flow cytometry (**B**) and statistical analysis (**C**) of MitoTracker Green staining in WT and SHP2$^{E76K}$ MSCs using flow cytometry (n=3 per group). Data are represented as the means ± SD. N.S. indicates no significance (two-tailed unpaired *t* test). **D** Western blot analysis of the expression of mitochondrial complex-associated proteins in WT and SHP2$^{E76K}$ MSCs.

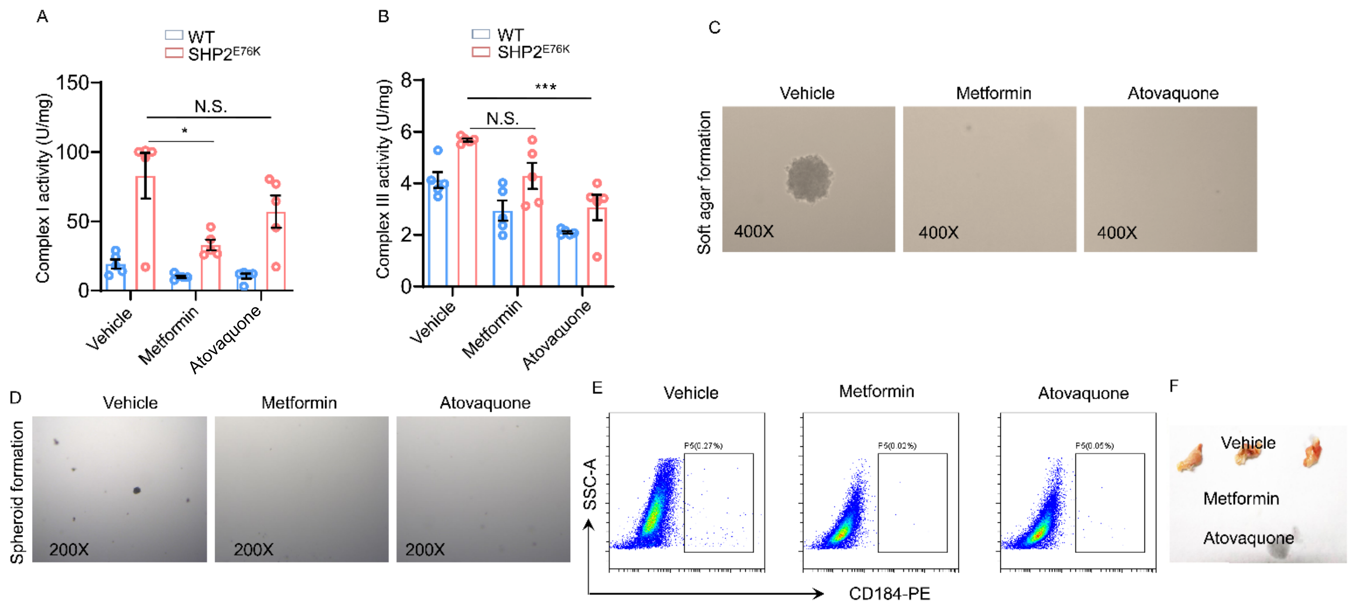


**Supplemental Figure 12. Metformin and atovaquone effectively inhibited the complex I and III activity and the population of CD184$^{+}$ cells in SHP2$^{E76K}$ MSCs. A, B** Statistical analysis of the activity of complexes I (**A**) and III (**B**) in WT and SHP2$^{E76K}$ MSCs following metformin and atovaquone treatment (n=5 per group). Data are represented as the means ± SD. **p* < 0.05, ****p* < 0.001, N.S. indicates no significance (Two-way ANOVA with multiple-comparison test). **C**, **D** Representative images of soft agar formation and spheroid formation of SHP2$^{E76K}$ MSCs with or without metformin and atovaquone treatment at indicated field. **E**, **F** Representative flow cytometry analysis of the population of CD184$^{+}$ cells and tumor formation of SHP2$^{E76K}$ MSCs with or without metformin and atovaquone treatment.

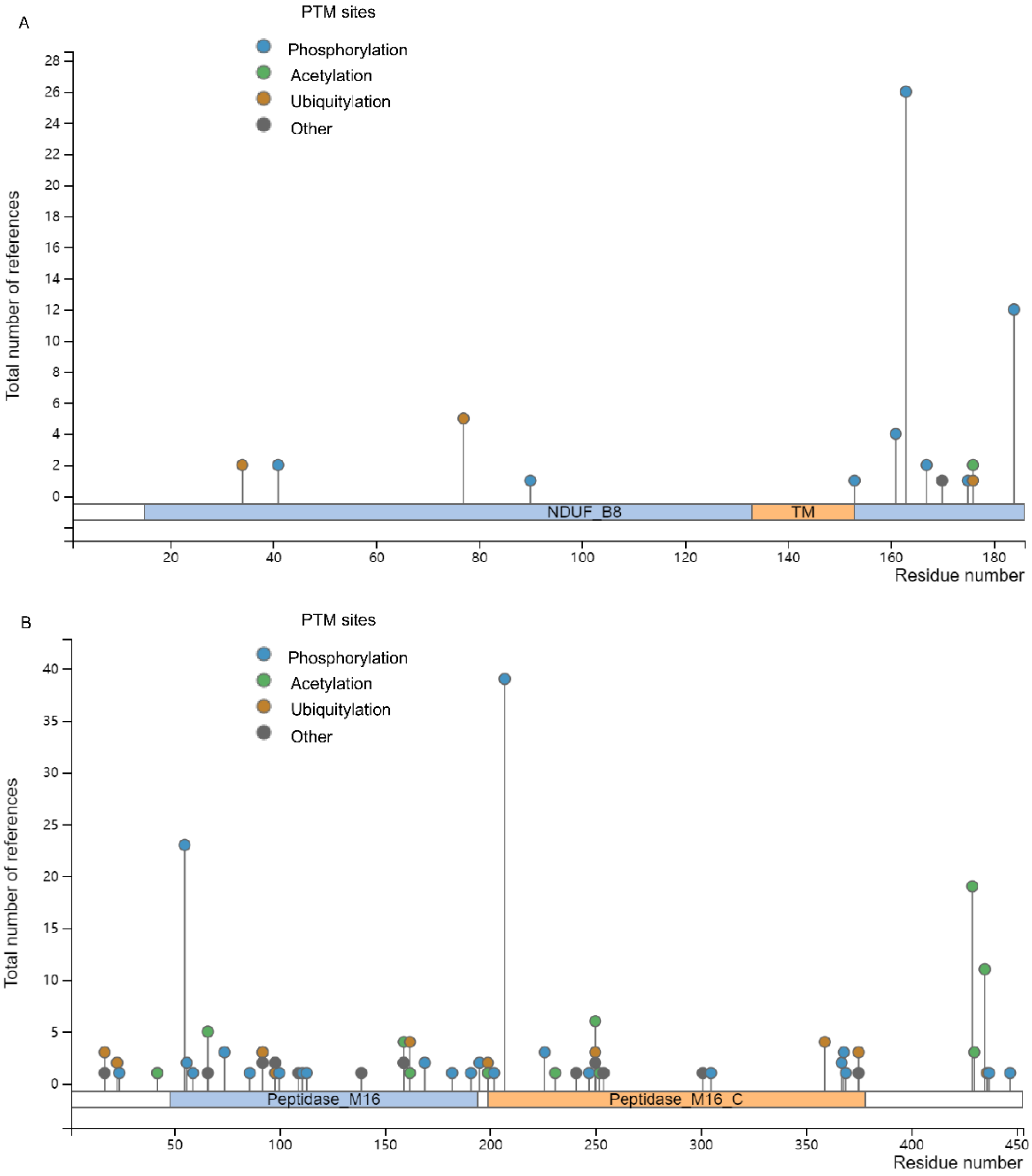


**Supplemental Figure 13. Protein posttranslational modification sites of NDUFB8 and UQCRC2. A** Protein posttranslational modification sites of NDUFB8, including phosphorylation, acetylation and ubiquitylation sites. **B** Protein posttranslational modification sites of UQCRC2, including phosphorylation, acetylation and ubiquitylation sites. Data are extracted from https://www.phosphosite.org.

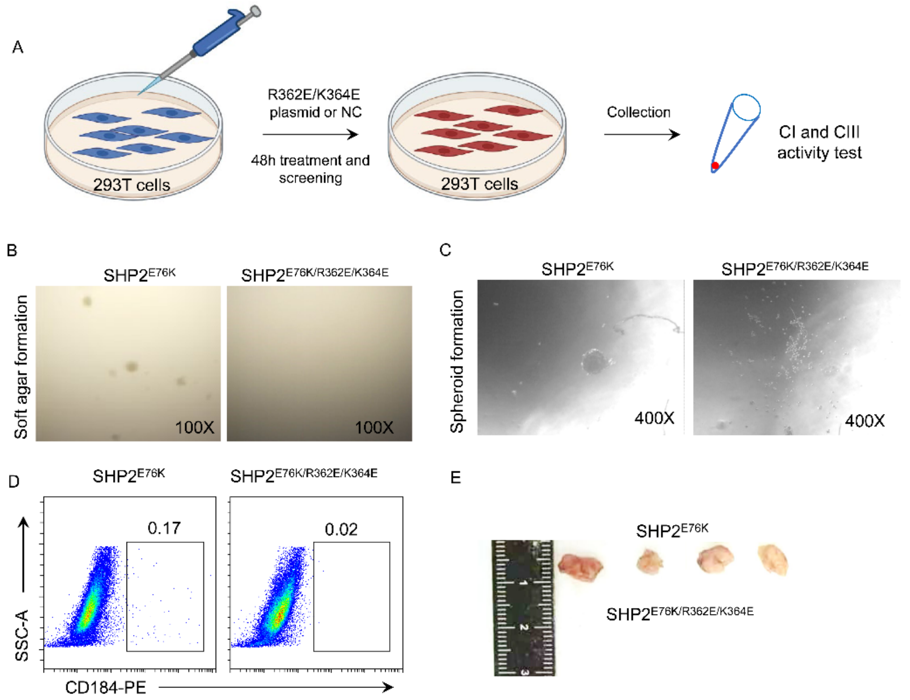


**Supplemental Figure 14. SHP2 LLPS-defective mutation rescues the malignancy of SHP2$^{E76K}$ MSCs.** **A** The Working flow that infection of SHP2 LLPS-defective mutation in 293T cells. **B, C** Representative soft agar images (**B**) and tumor spheroid formation (**C**) of SHP2$^{E76K}$ MSCs with or without R362E/K364E infection. **D, E** Representative flow cytometry analysis of CD184$^{+}$ cells (**D**) and tumor formation (**E**) of SHP2$^{E76K}$ MSCs infected with R362E/K364E plasmid.

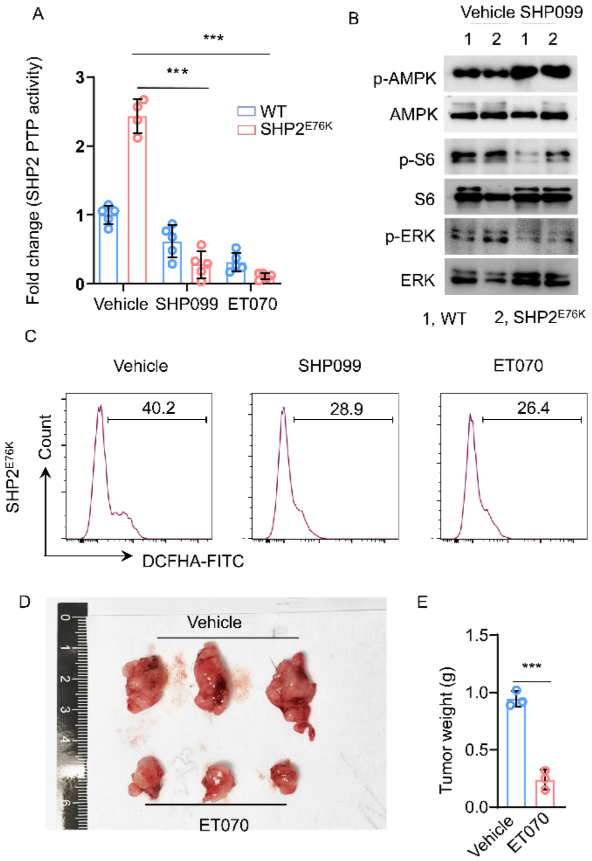


**Supplemental Figure 15. Alteration of SHP2 activity, downstream molecules and associated malignant cell behavior in SHP2$^{E76K}$ MSCs following treatment with SHP2 inhibitors. A** Statistical analysis of SHP2 activity in WT and SHP2$^{E76K}$ MSCs treated with SHP099 and ET070 (n= 5 per group). Data are represented as the means ± SD. **$p <$ 0.01 (Two-way ANOVA with multiple-comparison test). **B** Representative western blot images of the AMPK and ERK pathways in WT and SHP2$^{E76K}$ MSCs treated with SHP099 or ET070. **C** Representative flow cytometry images of ROS levels of SHP2$^{E76K}$ MSCs treated with SHP099 or ET070. Images were captured at a 200X field. **D**, **E** Representative tumor images (**D**) and statistical analysis (**E**) of tumor developed by SHP2$^{E76K}$ MSCs treated with ET070 (n=3 per group). Data are represented as the means ± SD. ***$p <$ 0.001 (two-tailed unpaired t test).
