# Supplementary material for: Phase separation of SHP2E76K promotes malignant transformation of mesenchymal stem cells by activating mitochondrial complexes
Source: JCI Insight. 2024 Mar 7;9(8):e170340. doi: 10.1172/jci.insight.170340 (PMC11141883; doi:10.1172/jci.insight.170340)

## Uncropped scans for Figure 6A

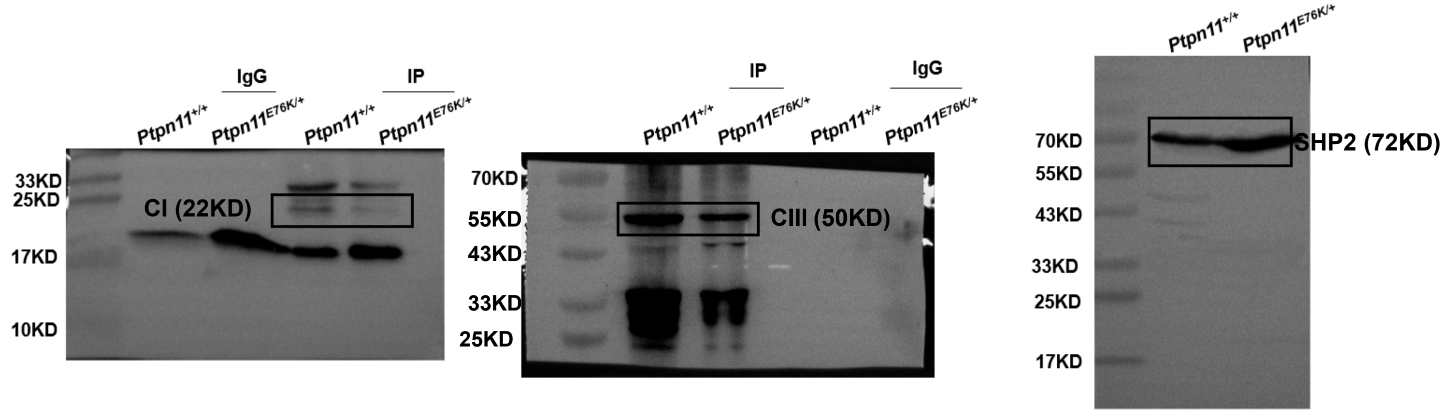


## Uncropped scans for Figure 6G

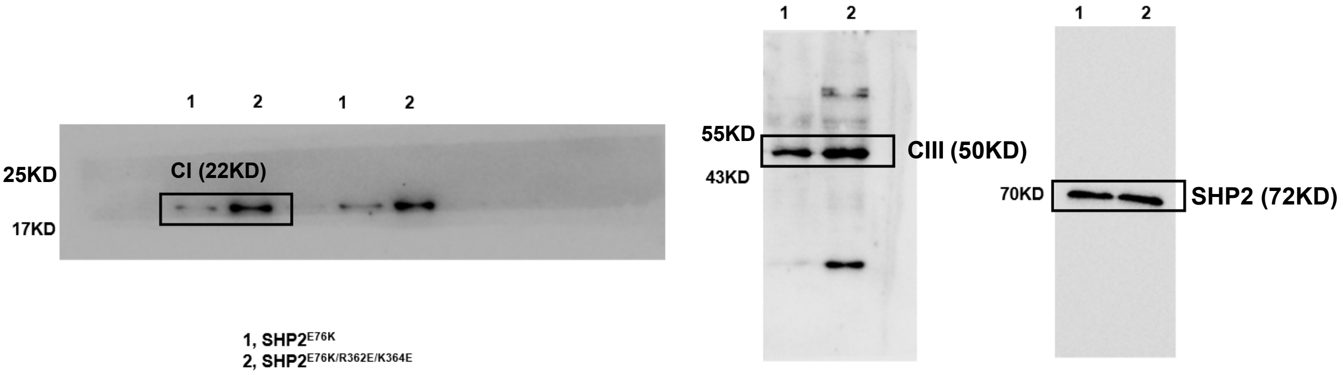


## Uncropped scans for Figure 7D

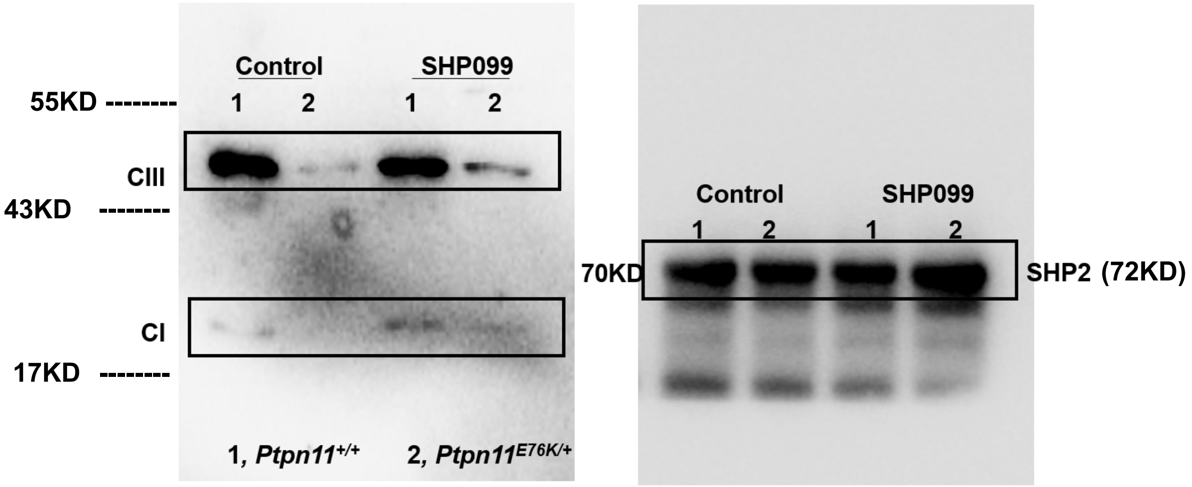

## Uncropped scans for Supplemental Figure 1D

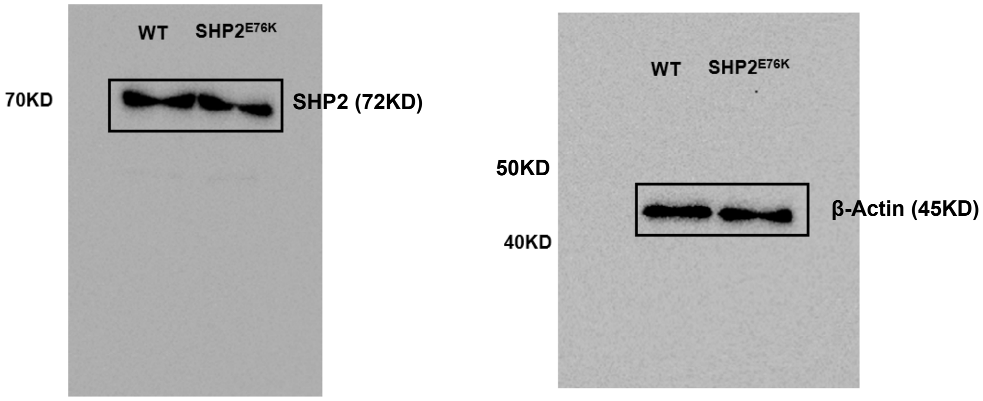


## Uncropped scans for Supplemental Figure 11D

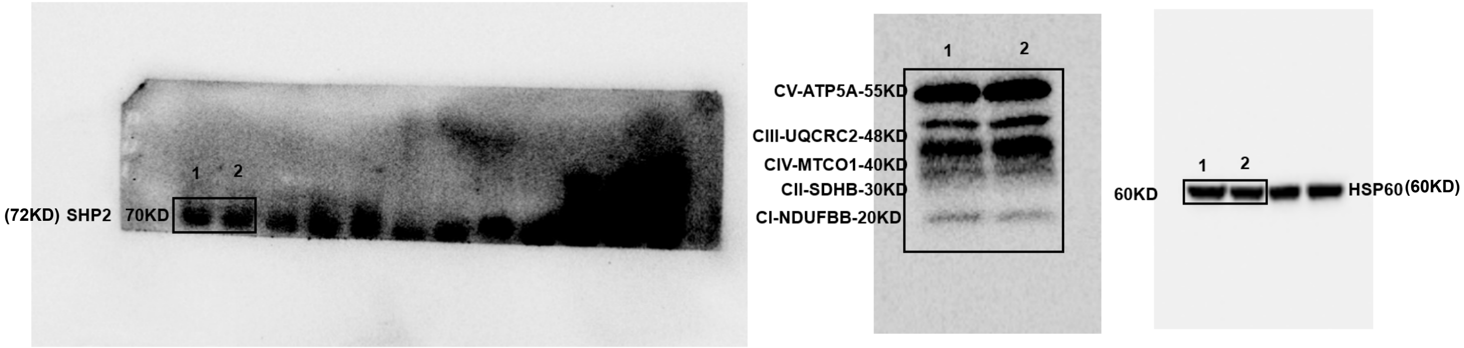


1, WT 2, SHP2$^{E76K}$

## Uncropped scans for Supplemental Figure 15B

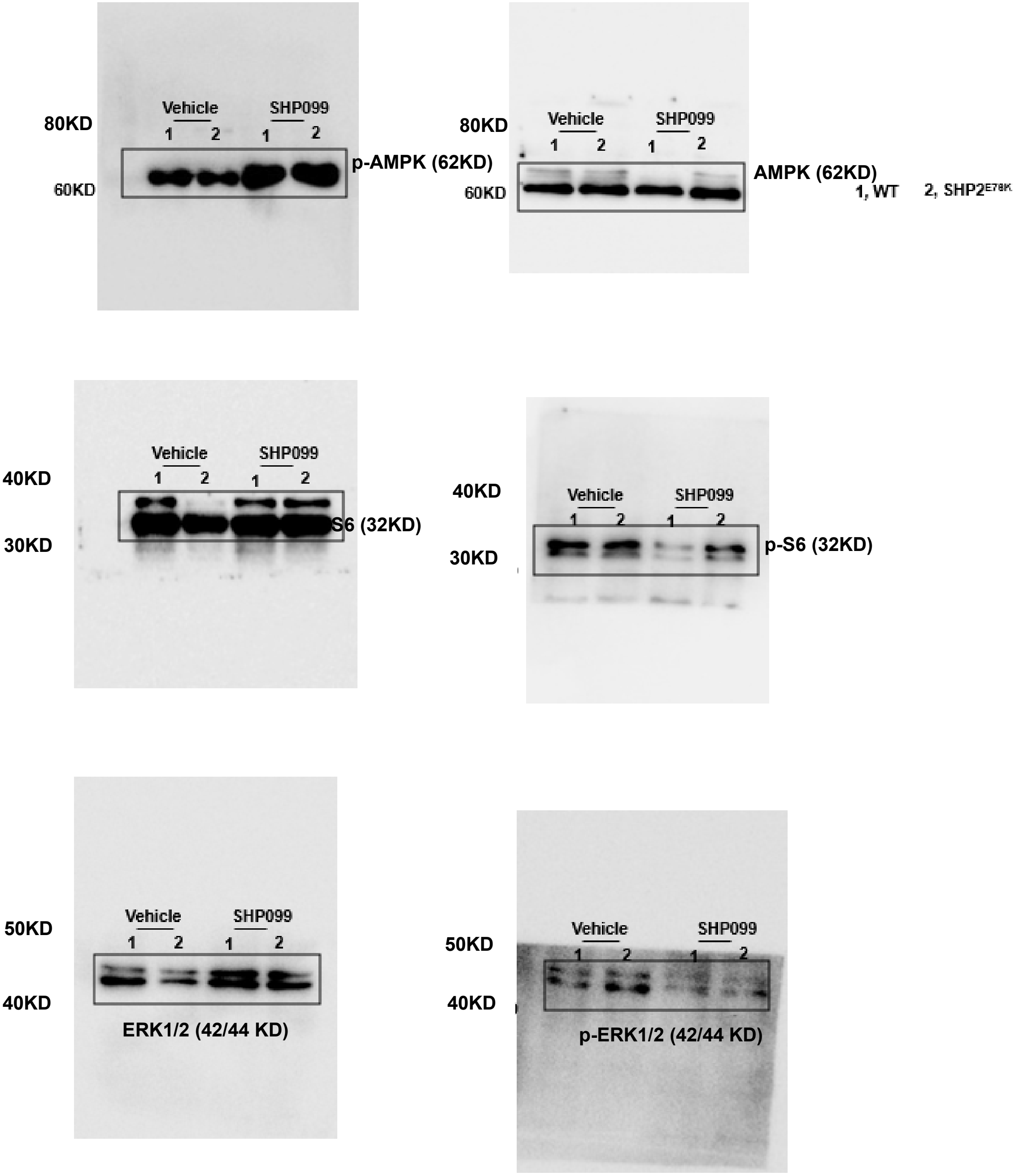

Supplement: Unedited blot and gel images [file jciinsight-9-170340-s009.pdf]
